# Synaptamide Ameliorates Hippocampal Neurodegeneration and Glial Activation in Mice with Traumatic Brain Injury

**DOI:** 10.3390/ijms241210014

**Published:** 2023-06-11

**Authors:** Anna Tyrtyshnaia, Olga Manzhulo, Igor Manzhulo

**Affiliations:** A.V. Zhirmunsky National Scientific Center of Marine Biology, Far Eastern Branch, Russian Academy of Sciences, Palchevskogo Str. 17, Vladivostok 690041, Russia; olga.manzhulo@bk.ru (O.M.);

**Keywords:** traumatic brain injury, synaptamide, N-docosahexaenoylethanolamine (DHEA), neuroinflammation, weight-drop injury model, hippocampus

## Abstract

Traumatic brain injury (TBI) is a major concern for public health worldwide, affecting 55 million people and being the leading cause of death and disability. To improve the outcomes and effectiveness of treatment for these patients, we conducted a study on the potential therapeutic use of N-docosahexaenoylethanolamine (synaptamide) in mice using the weight-drop injury (WDI) TBI model. Our study focused on exploring synaptamide’s effects on neurodegeneration processes and changes in neuronal and glial plasticity. Our findings showed that synaptamide could prevent TBI-associated working memory decline and neurodegenerative changes in the hippocampus, and it could alleviate decreased adult hippocampal neurogenesis. Furthermore, synaptamide regulated the production of astro- and microglial markers during TBI, promoting the anti-inflammatory transformation of the microglial phenotype. Additional effects of synaptamide in TBI include stimulating antioxidant and antiapoptotic defense, leading to the downregulation of the Bad pro-apoptotic marker. Our data suggest that synaptamide has promising potential as a therapeutic agent to prevent the long-term neurodegenerative consequences of TBI and improve the quality of life.

## 1. Introduction

The Lancet Commission on Neurology’s 2022 report reveals that traumatic brain injury (TBI) is a major global public health concern. TBI affects over 55 million people worldwide and is the leading cause of death and disability. The cost associated with TBI is a staggering USD 400 billion (GBP 350 billion) each year. TBI is not just an acute condition but also a chronic disease that increases the risk of developing neurodegenerative diseases such as dementia and Parkinson’s disease [1]. Although over 90% of TBI cases are classified as “mild,” more than half of these patients do not fully recover within six months of their injury. Therefore, improving treatment efficacy for these patients would be a significant public health benefit [2].

Effective treatment of TBI requires an integrated approach with a focus on mental health and long-term cognitive abnormalities. The complexity of the development mechanisms of TBI-associated mental disorders means that effective treatment requires an integrated approach that targets several pathogenesis links. Promising candidates for the treatment of TBI consequences are highly active lipid compounds called N-acylethanolamines of fatty acids (NAE) [3]. These endogenous metabolites and central nervous system (CNS) mediators possess anti-inflammatory, antioxidant, and neuroprotective properties.

This category of compounds comprises several types, such as N-docosahexaenoylethanolamine (DHEA), N-docosapentaenoylethanolamine (DPEA), N-eicosapentanoylethanolamine (EPEA), N-stearinoylethanolamine (STEA), N-arachidonoylethanolamine (AEA), N-palmitoylethanolamine (PEA), N-oleoylethanolamine (OEA), and others. N-arachidonoylethanolamine (AEA, anandamide), a metabolite of ω-6 arachidonic acid, affects the central nervous system primarily through CB receptor affinities. Therefore, it falls under the category of endocannabinoids, similar to 2-arachidonoylglycerol [4]. The activity of other fatty acid ethanolamides is mainly realized through cannabinoid-independent pathways. These pathways include the activation of the peroxisome-proliferator-activated nuclear receptor-α (PPAR-α), the downregulation of NF-kB and ERK1/2-dependent signaling, and the accumulation of cAMP followed by the phosphorylation of protein kinase A (PKA) and cAMP-response-element-binding protein (CREB) [5]. In the CNS, these compounds are metabolic intermediates and are synthesized “on demand”, acting as highly active mediators and regulators of physiological processes. A recent study shows that preventing the degradation of endogenous cannabinoid ligands after mild TBI attenuates neuroinflammation and improves the recovery of neurobehavioral function during the first 7 days after TBI induction [6]. Due to the neuritogenic and synaptogenic activity of DHEA, the substance was given the name “synaptamide” [7]. Synaptamide is an endogenous GPR110 (ADGRF1) ligand, the stimulation of which at nanomolar concentrations promotes axon growth and synaptogenesis and induces neuronal differentiation [8].

In this study, we explore the therapeutic potential of synaptamide in TBI using the weight-drop injury model (WDI). The effect of synaptamide on the processes of neurodegeneration and concomitant changes in neuronal and glial plasticity in the hippocampus of animals with TBI was studied. Data were obtained on the effect of synaptamide on TBI-associated behavioral changes, degeneration of the hippocampal neurons, the processes of glial activation, adult neurogenesis, cell apoptosis, and antioxidant protection in case of injury.

## 2. Results

### 2.1. The Impact of Synaptamide Treatment on Behavioral Changes in TBI

On the 6th and 7th days following surgery, we conducted behavioral tests to determine how the ventral and dorsal regions of the hippocampus were affected by TBI-related pathology. We also examined the impact of synaptamide treatment. One of the tests we used was the elevated plus maze, which helped us to evaluate anxiety-like behaviors that are modulated predominantly in the ventral hippocampus [9].

**Figure 1:**
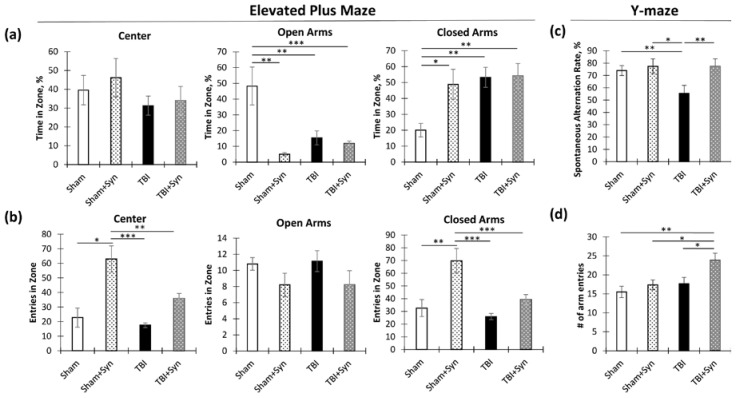
Behavioral changes in TBI and synaptamide treatment. (**a**) Percentage of time spent in the central, open, and closed areas of the elevated plus maze. (**b**) Number of entrances to the central, open, and closed areas of the elevated plus maze. (**c**) Spontaneous alternation rate in the Y-maze. (**d**) Number of entrances to the arms of the Y-maze. The results are shown as mean ± SEM of *n* = 10 independent animals. Two-way ANOVA followed by Tukey’s post hoc test (* *p* < 0.05, ** *p* < 0.01, *** *p* < 0.001).

In testing, we found that both TBI and treatment reduced the percentage of time spent in the open arms of the maze and increased the proportion of time spent in the closed arms. Moreover, a two-way analysis of variance in the percentage of time in open arms revealed a significant effect of an injury [F(1, 36) = 6.61; *p* = 0.014] and treatment [F(1, 36) = 21.34; *p* < 0.0001], as well as a significant effect of the interaction of these factors [F(1, 36) = 15.36; *p* = 0.0003]. Analysis of the percentage of time in open arms revealed a significant effect of an injury [F(1, 36) = 7.66; *p* = 0.008] and treatment [F(1, 36) = 4.54; *p* = 0.0039], as the effect of the interaction of these factors was [F(1, 36) = 4.03; *p* = 0.05]. These data indicated a significant effect of TBI on the degree of reduction in the percentage of time spent in open arms (Figure 1a). We concluded that synaptamide caused the sham-operated animals (“Sham + Syn”) to spend less time in open arms. However, in “TBI”, the number of entries was reduced compared to the “Sham” group, and synaptamide had no effect on this parameter. At the same time, the number of entrances to open arms did not change significantly either in TBI or after treatment, although there was a noticeable downward trend. However, the number of entrances to closed arms and the central part of the maze significantly increased under the action of synaptamide for the sham-operated animals (“Sham + Syn”) but not for the animals with TBI (“TBI + Syn”). A two-way analysis of variance in the number of entries into the central region revealed a significant effect of an injury [F(1, 36) = 8.89; *p* = 0.0049] and treatment [F(1, 36) = 20.23; *p* < 0.0001], as well as a significant effect of the interaction of these factors [F(1, 36) = 4.27; *p* = 0.045]. Similar effects were also found in the analysis of the number of entries into closed arms: injury [F(1, 36) = 9.75; *p* = 0.003] and treatment [F(1, 36) = 31.68; *p* < 0.0001], as well as a significant effect of the interaction of these factors [F(1, 36) = 4.47; *p* = 0.04] (Figure 1b). Thus, testing showed a decrease in the time spent in open arms and an increase in the time spent in closed arms both in sham-operated mice after the administration of synaptamide and in the animals with TBI, where synaptamide did not affect the result. At the same time, synaptamide increased the number of entrances to open arms and the central area of the maze only in the sham-operated animals, while TBI leveled this effect. This result is not amenable to unambiguous interpretation, since a decrease in the open/closed arms time ratio can mean both an increase in anxiety and a decrease in risk behavior.

To investigate the involvement of the dorsal hippocampus in TBI and assess the effects of synaptamide, we used Y-maze testing [10]. This test was performed to assess the working spatial memory, realized through the activity of the dorsal hippocampus. As a result, we found a decrease in the rate of spontaneous alternations in the TBI mice (“TBI”), with synaptamide treatment preventing the decrease (“TBI + Syn”). Two-way ANOVA revealed a significant treatment effect [F(1, 36) = 5.706; *p* = 0.022] and interactions of factors [F(1, 36) = 10.71; *p* = 0.002] (Figure 1c). In addition to studying working memory, the Y-maze was also used to determine locomotor activity. Two-way ANOVA revealed a significant effect of injury [F(1, 36) = 8.009; *p* = 0.007] and treatment [F(1, 36) = 6.824; *p* = 0.012] (Figure 1d). This result can be interpreted in such a way that synaptamide increased locomotor activity during trauma but did not affect the sham-operated animals.

### 2.2. Influence of TBI and Synaptamide Treatment on the Morphology of Hippocampal Neurons

**Figure 2:**
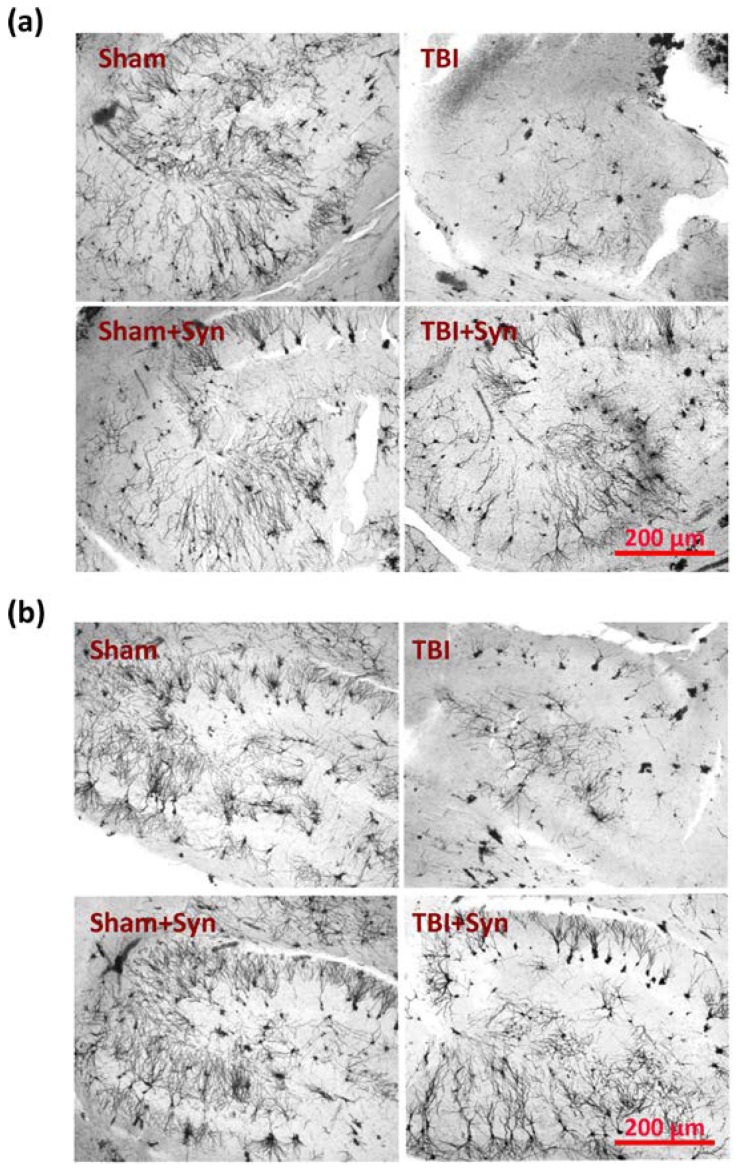
Representative overview images of the region of the lateral ventral (**a**) and dorsal (**b**) hippocampus of mice with TBI and treatment, where the analysis of the dendritic tree and dendritic spines of neurons was performed. Scale bar: 200 µm.

We conducted an analysis on the dendritic tree parameters in the CA1, CA3, and DG regions of the hippocampus to understand how trauma and treatment affect neurodegeneration. We marked the areas where the analysis of the dendritic tree and dendritic spines of neurons in TBI and treatment with synaptamide was performed by presenting overview photographs. In the study, micrographs of the ventral and dorsal regions of the lateral hippocampus were obtained and are presented in Figure 2. The medial region of the hippocampus was destroyed by TBI, but this was not the case for the lateral region. However, degenerative changes were observed in the neurons of the lateral region. This study focused specifically on the dorsal hippocampus, which is responsible for cognitive functions.

**Figure 3:**
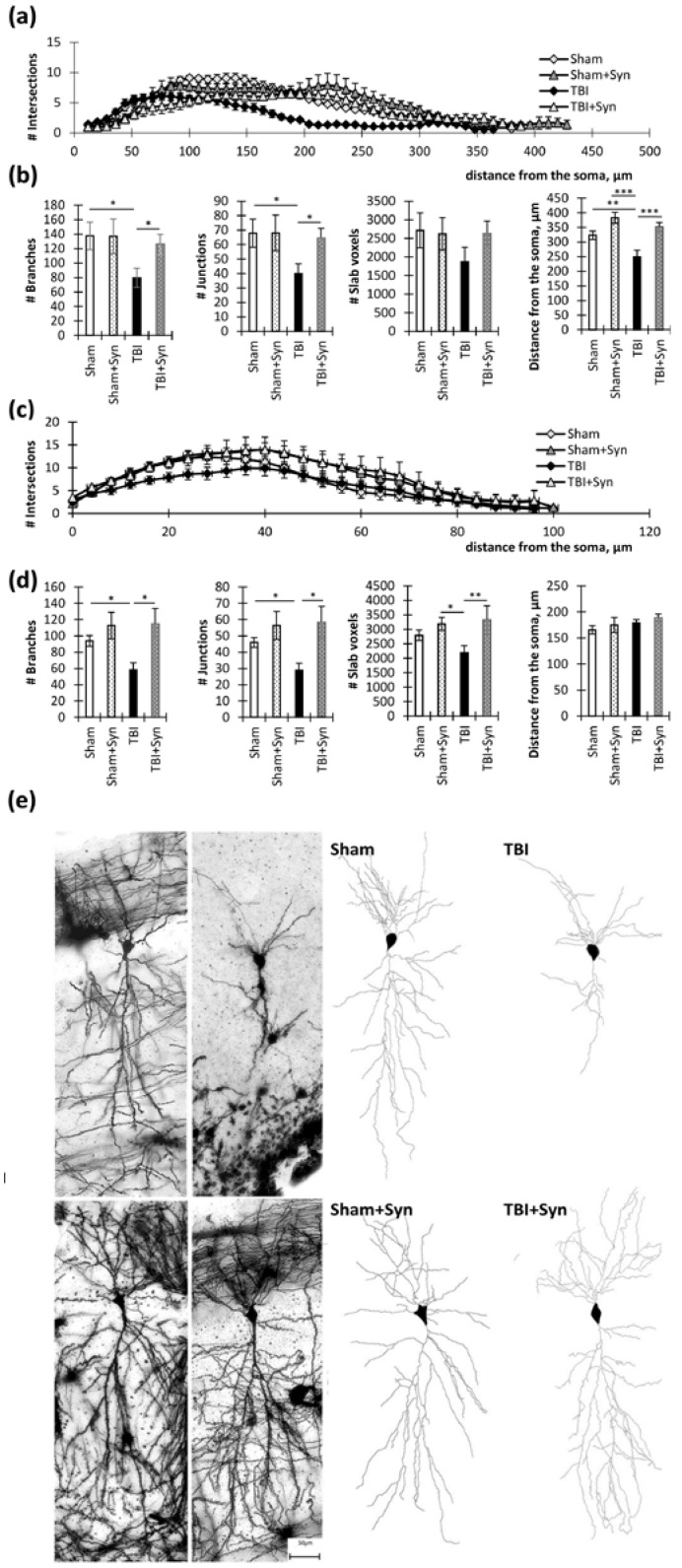
Hippocampal CA1 pyramidal neurons in TBI and synaptamide treatment. (**a**) The graph demonstrates the number of apical dendritic intersections depending on the distance from the soma. The results are shown as mean ± SEM of *n* = 12 independent neurons per group. (**b**) Dendritic tree analysis parameters of apical dendrites: total number of branches, total number of intersections, slab voxels, and distance from the soma. The results are shown as mean ± SEM of *n* = 12 independent neurons. Two-way ANOVA followed by Tukey’s post hoc test (* *p* < 0.05, ** *p* < 0.01, *** *p* < 0.001). (**c**) The graph demonstrates the number of basal dendritic intersections depending on the distance from the soma. The results are shown as mean ± SEM of *n* = 12 independent neurons per group. (**d**) Dendritic tree analysis parameters of basal dendrites: total number of branches, total number of intersections, slab voxels, and distance from the soma. The results are shown as mean ± SEM of *n* = 12 independent neurons (four animals per group, with three well-stained neurons obtained from one animal). Two-way ANOVA followed by Tukey’s post hoc test (* *p* < 0.05, ** *p* < 0.01). (**e**) Representative images of the CA1 hippocampal neurons of Golgi–Cox-stained sagittal brain sections. Scale bar: 50 µm.

In the apical dendrites of the CA1 region, there were significant differences in the number of intersections between the “TBI” and “Sham” groups at a distance of 80 to 270 µm from the soma (*p* < 0.05). Between the “TBI” and “Sham + Syn” groups, differences were present at a distance of 94 to 300 µm (*p* < 0.05). The “TBI” and “TBI + Syn” groups had the smallest difference interval, which was a distance from 136 to 282 µm (*p* < 0.05) (Figure 3a).

Two-way ANOVA of the total number of branches of the CA1 region apical dendrites revealed a significant effect of injury [F(1, 44) = 4.497; *p* = 0.0433], and a significant treatment effect was found [F(1, 44) = 4.25; *p* = 0.047]. Multiple comparisons revealed a significant reduction in the number of crossings in the “TBI” vs. “Sham” group (*p* = 0.03). A similar result was obtained in the analysis of the total number of intersections: a significant effect of injury [F(1, 44) = 4.323; *p* = 0.0476] and a significant treatment effect were found [F(1, 44) = 0.196; *p* = 0.047]. However, analysis of the maximum distances of dendrites from the center of the soma revealed a significant effect of both injury [F(1, 44) = 10.07, *p* = 0.0036] and treatment [F(1, 44) = 10.07; *p* = 0.0036] (Figure 3b).

In the basal dendrites of the CA1 region, there were significant differences in the number of intersections between the “TBI” and “Sham” groups at a distance of 36 to 70 µm from the soma (*p* < 0.05). Between the “TBI” and “Sham + Syn” groups, differences were present at a distance of 38 to 84 µm (*p* < 0.05). The “TBI” and “TBI + Syn” groups did not have significant differences in the number of intersections (Figure 3c).

Two-way analysis of variance of the total number of branching of the basal dendrites of the CA1 region revealed a significant treatment effect [F(1, 44) = 14.79; *p* = 0.0004], but there was no significant effect of injury [F(1, 44) = 2.114; *p* = 0.1531]. A similar result was obtained in the analysis of the slab voxels parameter, where a significant effect of the treatment was revealed [F(1, 38) = 9.515; *p* = 0.0038], and there was no effect of injury [F(1, 44) = 1.84; *p* = 0.1821]. Analysis of the total length of the dendrites demonstrated no significant differences between the groups (Figure 3d). Representative images of neurons are shown in Figure 3e.

**Figure 4:**
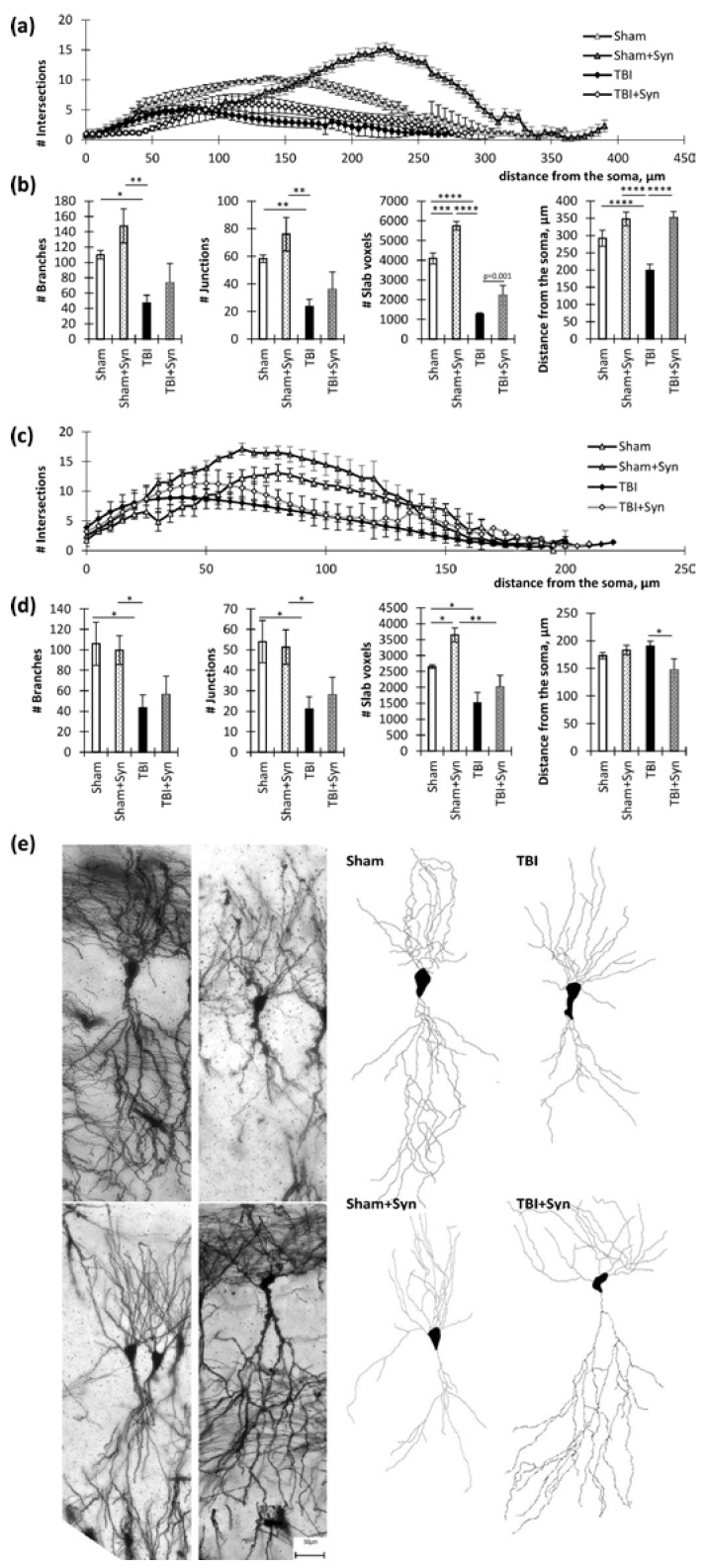
Hippocampal CA3 pyramidal neurons in TBI and synaptamide treatment. (**a**) The graph demonstrates the number of apical dendritic intersections depending on the distance from the soma. The results are shown as mean ± SEM of *n* = 12 independent neurons per group. (**b**) Dendritic tree analysis parameters of apical dendrites: total number of branches, total number of intersections, slab voxels, and distance from the soma. The results are shown as mean ± SEM of *n* = 12 independent neurons. Two-way ANOVA followed by Tukey’s post hoc test (* *p* < 0.05, ** *p* < 0.01, *** *p* < 0.001, **** *p* < 0.0001). (**c**) The graph demonstrates the number of basal dendritic intersections depending on the distance from the soma. The results are shown as mean ± SEM of *n* = 12 independent neurons per group (four animals per group, with three well-stained neurons obtained from one animal). (**d**) Dendritic tree analysis parameters of basal dendrites: total number of branches, total number of intersections, slab voxels, and distance from the soma. The results are shown as mean ± SEM of *n* = 12 independent neurons. Two-way ANOVA followed by Tukey’s post hoc test (* *p* < 0.05, ** *p* < 0.01). (**e**) Representative images of the CA1 hippocampal neurons of Golgi–Cox-stained sagittal brain sections. Scale bar: 50 µm.

To the greatest extent, the changes affected the dendritic morphology in the CA3 region. In the apical dendrites of the CA3 region, there were significant differences in the number of intersections between the “TBI” and “Sham” groups at a distance of 75 to 240 µm from the soma (*p* < 0.05). Between the “TBI” and “Sham + Syn” groups, differences were present at a distance of 105 to 255 µm (*p* < 0.05). The “TBI” and “TBI + Syn” groups had the smallest difference interval, which was a distance from 5 to 85 µm (*p* < 0.05) (Figure 4a).

Two-way ANOVA of the total number of branches of the CA1 region apical dendrites revealed a significant effect of injury [F(1, 44) = 17.92; *p* = 0.0008], but there was no significant treatment effect [F(1, 44) = 9.904; *p* = 0.068]. Multiple comparisons revealed a significant reduction in the number of intersections in the “TBI” vs. “Sham” group (*p* = 0.004). Slab voxels analysis reflecting the total length of the dendrites demonstrated a significant effect of an injury [F(1, 44) = 111.5; *p* < 0.0001] and treatment [F(1, 28) = 18.77; *p* = 0.0007] for this parameter. At the same time, trauma significantly reduced the total length of dendrites (*p* < 0.001), and the drug administered to sham-operated animals increased this parameter (*p* < 0.001). However, analysis of the mean maximum distance of dendrites from the center of the soma revealed a significant effect of both injuries [F(1, 44) = 6.43, *p* = 0.0213] and treatment [F(1, 44) = 35.54; *p* < 0.0001], as well as their interactions [F(1, 44) = 7.64, *p* = 0.0133]. This indicated a favorable effect of the drug on the dendritic length. Multiple comparisons revealed a significant reduction in the distance from the soma in the injury animals compared to the controls (292.00 ± 23.08 in “Sham” vs. 199.00 ± 17.28 in “TBI”, *p* = 0.006), while the value in the “TBI + Syn” group was significantly higher than in the “TBI” group (352 ± 17.19 in “TBI + Syn”, *p* < 0.0001 compared to “TBI”) (Figure 4b).

In the basal dendrites of the CA3 region, there were significant differences in the number of intersections between the “TBI” and “Sham” groups at a distance of 65 to 155 µm from the soma (*p* < 0.05). Between the “TBI” and “Sham + Syn” groups, differences were present at a distance of 55 to 150 µm (*p* < 0.05). The “TBI” and “TBI + Syn” groups had no significant differences in the number of intersections (Figure 4c). Two-way ANOVA of the total number of branching of the CA3 region basal dendrites revealed a significant effect of injury [F(1, 44) = 13.65; *p* = 0.0027]; however, no significant treatment effect was found [F(1, 44) = 0.05; *p* = 0.81]. A similar result was obtained in the analysis of the slab voxels, where a significant effect of trauma was revealed [F(1, 44) = 8.972; *p* = 0.01]. In addition, an effect of the interaction of the two studied factors was found [F(1, 44) = 30.97; *p* < 0.0001]. It was in the absence of trauma when the drug was injected that we observed a significant increase in the number of slab voxels compared to the control (2652.00 ± 54.48 in “Sham” vs. 3646.00 ± 216.37 in “TBI”, *p* = 0.02). When analyzing the length of dendrites, expressed as the distance from the soma, we found a significant effect of the interaction of the studied factors [F(1, 44) = 5.88; *p* = 0.02]. Thus, it was in trauma that we observed a reducing effect of the drug on this parameter (190.00 ± 9.38 vs. 147 ± 20.43, *p* = 0.049) (Figure 4d). Representative images of neurons are shown in Figure 4e.

**Figure 5:**
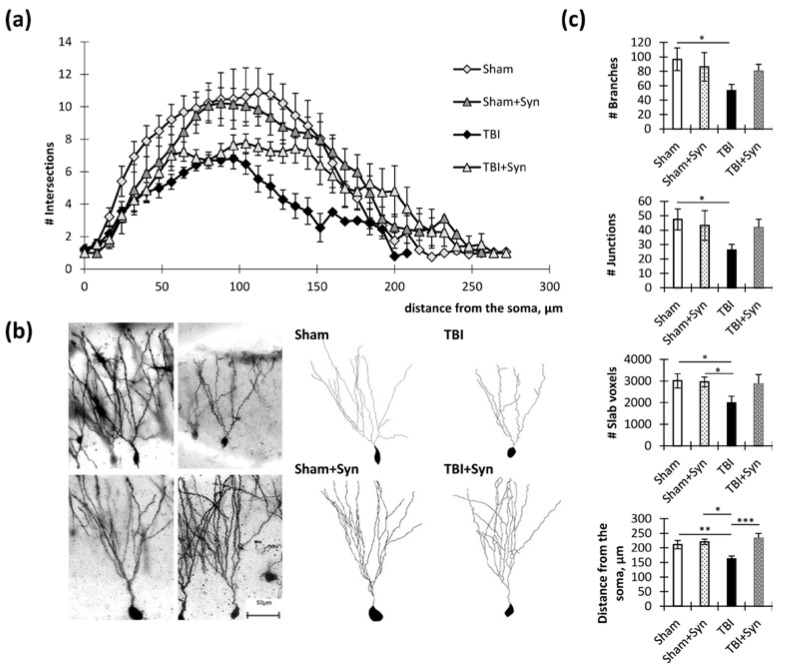
Hippocampal DG granular neurons in TBI and synaptamide treatment. (**a**) The graph demonstrates the number of dendritic intersections depending on the distance from the soma. The results are shown as mean ± SEM of *n* = 12 independent neurons per group. (**b**) Representative images of the DG granular neurons of Golgi–Cox-stained sagittal brain sections. Scale bar: 50 µm. (**c**). Dendritic tree analysis parameters of apical dendrites: total number of branches, total number of intersections, slab voxels, and distance from the soma. The results are shown as mean ± SEM of *n* = 12 independent neurons (four animals per group, with three well-stained neurons obtained from one animal). Two-way ANOVA followed by Tukey’s post hoc test (* *p* < 0.05, ** *p* < 0.01, *** *p* < 0.001).

In the dendrites of DG granular neurons, there were significant differences in the number of intersections between the “TBI” and “Sham” groups at a distance of 32 to 152 µm from the soma (*p* < 0.05). Between the “TBI” and “Sham + Syn” groups, differences were present at a distance of 64 to 156 µm (*p* < 0.05). The “TBI” and “TBI + Syn” groups had the smallest difference interval, which was a distance from 120 to 152 µm (*p* < 0.05) (Figure 5a). Representative images of neurons are shown in Figure 5b.

Two-way ANOVA of the total number of branches and number of junctions of the DG region revealed a significant effect of injury [F(1, 44) = 11.26; *p* = 0.0014], but there was no significant treatment effect in both parameters. Slab voxels analysis demonstrated a significant effect of an injury [F(1, 44) = 10.65; *p* = 0.0019] and treatment [F(1, 28) = 6.17; *p* = 0.0015] for this parameter. In addition, the analysis revealed a significant effect of treatment on the dendritic length, expressed as the maximum distance from the center of the soma [F(1, 44) = 11.91; *p* = 0.0021]. In addition, an effect of injury [F(1, 44) = 5.46; *p* = 0.023] as well as interaction effect was found [F(1, 44) = 6.802; *p* = 0.0154] (Figure 5c).

Thus, the statistical analysis made it possible to draw several conclusions regarding the effect of synaptamide on the morphology of hippocampal dendrites. In the CA1, CA3, and DG regions, synaptamide interfered with the TBI-associated decrease in the number of apical dendritic intersections in a certain range of distances from the soma. Treatment with synaptamide prevented the TBI-mediated decrease in the mean maximum distance from the soma of the CA1 region apical dendrites and DG granular neuron dendrites. In the CA3 region, which was the most damaged during trauma, the drug did not affect this parameter. In addition, synaptamide increased the total number of branches in the basal dendrites of the CA1 region.

### 2.3. The Impact of Trauma and Treatment on Dendritic Spine Morphology

**Figure 6:**
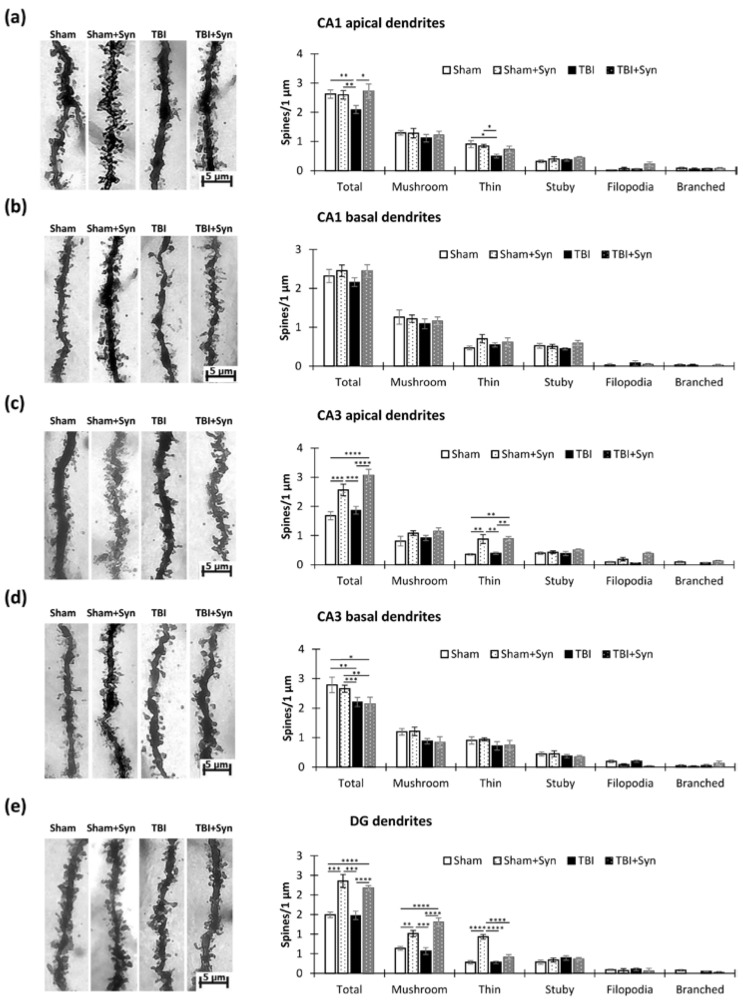
Dendritic spines in the hippocampus CA1, CA3, and DG neurons during TBI and synaptamide treatment were visualized by Golgi–Cox staining. (**a**) Representative images demonstrating dendritic spines in the apical dendrites of hippocampal CA1 pyramidal neurons (**left**) and spine number quantification per 1 µm (**right**), * *p* < 0.05, ** *p* < 0.01. (**b**) Representative images demonstrating dendritic spines in the basal dendrites of hippocampal CA1 pyramidal neurons (left) and spine number quantification per 1 µm (**right**). (**c**) Representative images demonstrating dendritic spines in the apical dendrites of hippocampal CA3 pyramidal neurons (left) and spine number quantification per 1 µm (**right**), ** *p* < 0.01, *** *p* < 0.001, **** *p* < 0.0001. (**d**) Representative images demonstrating dendritic spines in the basal dendrites of hippocampal CA3 pyramidal neurons (**left**) and spine number quantification per 1 µm (**right**), * *p* < 0.05, ** *p* < 0.01, *** *p* < 0.001. (**e**) Representative images demonstrating dendritic spines in the dendrites of hippocampal DG granular neurons (**left**) and spine number quantification per 1 µm (**right**), ** *p* < 0.01, *** *p* < 0.001, **** *p* < 0.0001. The results are shown as mean ± SEM of *n* = 12 independent neurons (four animals per group, with three well-stained neurons obtained from one animal). Two-way ANOVA followed by Tukey’s post hoc test.

Quantitative analysis of dendritic spines revealed a significant decrease in the total number of spines during trauma in the CA1 region apical dendrites (2.62 ± 0.14—“Sham” vs. 2.09 ± 0.13—“TBI”, *p* < 0.01). This decrease occurred mainly due to thin spines (0.90 ± 0.12—“Sham” vs. 0.49 ± 0.06—“TBI”, *p* < 0.05), and the ratio of mushroom spines did not change as a result of injury. Treatment with synaptamide prevented a decrease in the total spine number and number of thin spines. Two-way ANOVA revealed an effect for both injury [F(1, 32) = 4.66.1; *p* = 0.049] and treatment [F(1, 44) = 5.74; *p* = 0.01] (Figure 6a). The basal spines did not undergo quantitative changes either during trauma or during treatment (Figure 6b).

In the CA3 region, trauma did not affect the number of spines on the apical dendrites; however, the drug administration significantly increased the density of the spines compared to the control (1.67 ± 0.13—“Sham” vs. 2.56 ± 0.20—“Sham + Syn”, *p* < 0.001). As in the case of the CA1 region, the increase occurred mainly due to changes in the number of thin spines (0.35 ± 0.02—“Sham” vs. 087 ± 0.15—“Sham + Syn”, *p* < 0.01). Two-way ANOVA revealed a significant treatment effect [F(1, 44) = 366.1; *p* < 0.0001] (Figure 6c). When analyzing the basal spines of the CA3 region, we found that trauma significantly reduced the total number of spines (2.78 ± 0.26—“Sham” vs. 2.20 ± 0.15—“TBI”, *p* < 0.001), but the treatment did not affect their number. Two-way ANOVA revealed a significant effect of injury [F(1, 44) = 9.306; *p* = 0.0057] (Figure 6d).

In the DG region, we did not find an effect of trauma on the density of spines; however, the drug administration significantly increased this parameter (1.49 ± 0.07—“Sham” vs. 2.35 ± 0.16—“Sham + Syn”, *p* < 0.0001). An increase in the density of spines occurred both due to mushroom-shaped (0.63 ± 0.04—“Sham” vs. 1.01 ± 0.7—“Sham + Syn”, *p* < 0.01) and thin spines (0.28 ± 0.04—“Sham” vs. 0.93 ± 0.05—“Sham + Syn”, *p* < 0.0001). In the mushroom spines, an increase in density was observed in the synaptamide-treated TBI group compared to the “TBI” group (*p* < 0.0001). Two-way ANOVA of the total spine number revealed a significant treatment effect [F(1, 44) = 57.40; *p* < 0.0001] (Figure 6e).

Thus, we concluded that in the CA1 region dendrites, synaptamide was effective in preventing the TBI-associated reduction in spine density. At the same time, in the CA3 and DG regions, the drug increased the density of spines, regardless of the presence of TBI. The injury itself had no significant effect on the density of spines in the CA3 and DG regions except for the basal dendrites of the CA3 region.

### 2.4. The Effect of TBI and Synaptamide Treatment on the State of Hippocampal Microglia and the Production of Pro- and Anti-Inflammatory Factors

**Figure 7:**
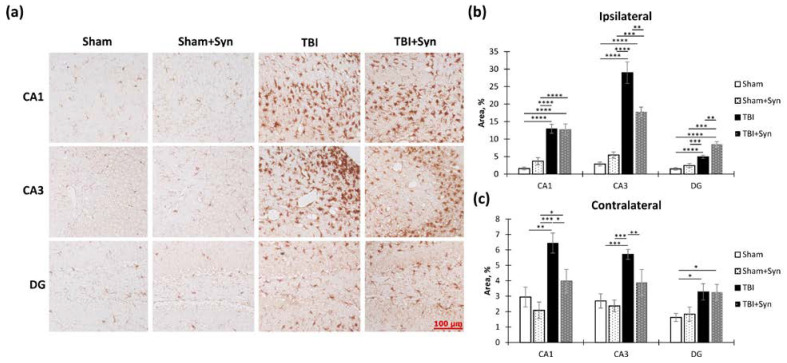
Iba-1 immunoreactivity within the CA1, CA3, and DG hippocampal areas in TBI and synaptamide treatment groups. (**a**) Representative photomicrographs of Iba-1-positive stained CA1, CA3, and DG areas of ipsilateral hippocampal coronal sections. Scale bar: 100 µm. (**b**) Iba-1-positive stained area in CA1, CA3, and DG areas of the ipsilateral hippocampus, %. (**c**) Iba-1-positive stained area in CA1, CA3, and DG areas of the contralateral hippocampus, %. The results are shown as mean ± SEM of *n* = 10 independent slices. Two-way ANOVA followed by Tukey’s post hoc test (* *p* < 0.05, ** *p* < 0.01, *** *p* < 0.001, **** *p* < 0.0001).

In order to explore the effects of TBI and treatment on microglial activation within the hippocampus, we performed an immunohistochemical analysis using the microglial marker Iba-1. Specifically, we examined the ipsi- and contralateral hippocampus. The accompanying Figure 7a displays sample photomicrographs of Iba-1-positive stained CA1, CA3, and DG regions of the ipsilateral hippocampal coronal sections.

We found that in the ipsilateral hippocampus, injury significantly increased Iba-1 immunoreactivity in the CA1 (1.59 ± 0.41—“Sham” vs. 12.94 ± 1.28—“TBI”, *p* < 0.0001), CA3 (2.84 ± 0.57—“Sham” vs. 28.95 ± 3.07—“TBI”, *p* < 0.0001) and DG (1.47 ± 0.36—“Sham” vs. 4.92 ± 0.45—“TBI”, *p* < 0.0001) regions. Two-way ANOVA revealed a significant effect of injury [F(1, 36) = 82.30; *p* < 0.0001]; however, no treatment effect was observed. In the CA3 region, two-way ANOVA revealed a significant effect of injury (F(1, 36) = 102.4; *p* < 0.0001) and treatment [F(1, 36) = 5.104; *p* = 0.0293], and it identified a significant interaction between injury and treatment [F(1, 36 = 13.15; *p* = 0.0008]. This result indicated that synaptamide prevented the development of a TBI-mediated increase in Iba-1 immunoreactivity. A significant effect of injury was found in the DG region [F(1, 36) = 102.40, *p* < 0.0001], as was an effect of treatment [F(1, 36) = 5.104, *p* = 0.029,] and interaction [F(1, 36) = 13.15, *p* = 0.0008]. This result reflected the dynamics of the changes in Iba-1 immunoreactivity, since in the mice with TBI, synaptamide increased Iba-1 immunoreactivity by almost two times (4.92 ± 0.45—“TBI” vs. 8.47 ± 0.76—“TBI + Syn”, *p* = 0.0011) (Figure 7b). In the contralateral hippocampus, changes in Iba-1 immunoreactivity were not so pronounced; however, in general, the directions of the changes were similar to those in the ipsilateral hippocampus. In the CA1 and CA3 regions, synaptamide prevented an increase in TBI-mediated immunoreactivity (Figure 7c).

**Figure 8:**
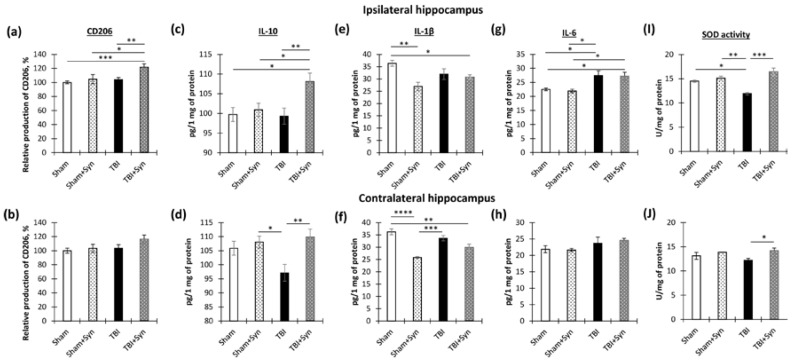
Effect of synaptamide on TBI-induced production of pro- and anti-inflammatory factors, and superoxide dismutase (SOD) activity within the hippocampus. (**a**) CD-206 production within the ipsilateral hippocampus, %. (**b**) CD-206 production within the contralateral hippocampus, %. (**c**) IL-10 production within the ipsilateral hippocampus. (**d**) IL-10 production within the contralateral hippocampus, pg/1 mg of protein. (**e**) IL-1β production within the ipsilateral hippocampus, pg/1 mg of protein. (**f**) IL-1β production within the contralateral hippocampus, pg/1 mg of protein. (**g**) IL-6 production within the ipsilateral hippocampus, pg/1 mg of protein. (**h**) IL-6 production within the contralateral hippocampus, pg/1 mg of protein. (**I**) SOD activity within the ipsilateral hippocampus. (**J**) SOD activity within the contralateral hippocampus. The results are shown as mean ± SEM of *n* = ten independent samples (five animals per group, with two samples per one animal). Two-way ANOVA followed by Tukey’s post hoc test (* *p* < 0.05, ** *p* < 0.01, *** *p* < 0.001, **** *p* < 0.0001).

We conducted a study to explore the impact of synaptamide on the Iba1-immunoreactivity in the hippocampus. Our focus was on the production of pro-inflammatory cytokines (IL-1β, IL-6) and anti-inflammatory factors (IL-10, CD206) within the hippocampus using ELISA. Apart from cytokines, we also examined CD206 immunoreactivity, a protein commonly found in the M2 (anti-inflammatory) macrophage population, as an anti-inflammatory marker [11]. Two-way ANOVA of the changes in CD206 dynamics in the ipsilateral hippocampus demonstrated a significant effect of injury [F(1, 37) = 7.22; *p* = 0.01] and treatment [F(1, 37) = 7.865; *p* = 0.0075]. This result reflected the fact that an increase in CD206 production occurred only in the synaptamide-treated TBI animals (104.07 ± 2.75%—“TBI” vs. 121.65 ± 4.82%—“TBI + Syn”, *p* = 0.006) (Figure 8a). In the contralateral hippocampus, we did not find statistically significant changes in the production (Figure 8b). We observed a similar result for the anti-inflammatory cytokine IL-10 in the ipsilateral hippocampus; two-way ANOVA revealed a significant treatment effect [F(1, 37) = 6.894; *p* = 0.0116]. In the synaptamide-treated TBI animals, there was an increase in IL-10 production (99.28 ± 2.05 pg/mg of protein—“TBI” vs. 108.05 ± 2.18 pg/mg of protein—“TBI + Syn”, *p* = 0.0072) (Figure 8c). In the contralateral hippocampus, the analysis revealed a significant treatment effect [F(1, 37) = 8.22; *p* = 0.006] and an injury/treatment interaction [F(1, 37) = 4.102; *p* = 0.048] (Figure 8d). This suggested that in the untreated animals, TBI caused a decrease in IL-10 production, while treatment with synaptamide prevented such a decrease. An increase in CD206 and IL-10 production with a simultaneous increase in Iba-1 immunoreactivity in the synaptamide-treated TBI animals may indicate a stimulating effect of synaptamide on the polarization of microglia towards the M2 phenotype.

At the same time, in the sham-operated animals, synaptamide administration caused a decrease in the production of the pro-inflammatory cytokine IL-1β (36.37 ± 1.18 pg/mg of protein—“TBI” vs. 26.94 ± 1.71 pg/mg of protein—“TBI + Syn”, *p* = 0.0019). A significant treatment effect [F(1, 37) = 11.31; *p* = 0.0016] and an injury/treatment interaction [F(1, 37) = 6.78; *p* = 0.0125] were found (Figure 8e). A similar effect was also found in the contralateral hippocampus, where the treatment effect was [F(1, 37) = 36.12; *p* < 0.0001], and the effect of the factors’ interaction was [F(1, 37) = 7.88; *p* = 0.0074] (Figure 8f). This result indicated that synaptamide treatment reduced the production of the pro-inflammatory cytokine IL-1β in the hippocampus, but this decrease was affected by the presence or absence of trauma. Thus, in the absence of injury, we observed a more pronounced decrease, although a significant decrease in IL-1β production was also present in the TBI animals.

At the same time, the production of another pro-inflammatory cytokine, IL-6, changed to a different kind. Thus, TBI increased the production of IL-6, and treatment with synaptamide did not interfere with this effect. Two-way ANOVA revealed a significant effect of injury [F(1, 37) = 15.95; *p* = 0.0002] and no treatment effect. These results explained the increase in Iba-1 immunoreactivity in the “TBI” group (Figure 8g). At the same time, we did not find statistically significant differences in the contralateral hippocampus (Figure 8h).

Thus, TBI is accompanied by a dynamic change in the production of pro- and anti-inflammatory cytokines and M1-microglia polarization. The data obtained indicate that synaptamide in TBI promotes M2 polarization of microglia, with a shift in the profile of secreted cytokines towards the anti-inflammatory side.

Following TBI, microglia are quickly activated, producing high levels of ROS via mitochondrial and NADPH oxidase pathways, damaging neurons and glial cells due to oxidative stress. Oxidative stress, which occurs when there is an imbalance between the production of reactive oxygen species (ROS) and antioxidant enzymes, is known to play a significant role in secondary neuronal damage in TBI. A maladaptive increase in ROS production leads to neuronal damage and apoptotic cell death [12]. In this study, we evaluated the activity of the antioxidant enzyme superoxide dismutase (SOD) in order to assess the antioxidant capacity of synaptamide.

In the study of SOD activity in the ipsilateral hippocampus, we found a stimulating effect of synaptamide on the production of this enzyme. Two-way ANOVA showed a significant effect of treatment [F(1, 36) = 11.65; *p* = 0.003] and an injury/treatment interaction of injury factors [F(1, 36) = 7.02; *p* = 0.017] (Figure 8I). In the contralateral hippocampus, two-way ANOVA revealed a significant effect of treatment [F(1, 36) = 8.38; *p* = 0.01] (Figure 8J).

### 2.5. The Effect of TBI and Synaptamide Treatment on the State of Hippocampal Astroglia

Astrocytes play a crucial role in the brain, as they are involved in the formation of the blood–brain barrier, maintaining ion balance, and regulating energy metabolism. However, in cases of traumatic brain injury, astrocytes can have a dual effect. On the one hand, they can form a protective astroglial scar that prevents further damage to other parts of the brain. On the other hand, altered astrocytes may release excessive amounts of excitatory amino acids, leading to excitotoxicity and neuronal loss. To better understand how astrocytes change during trauma and treatment, we utilized astrocytic markers GFAP and vimentin in our studies [13].

**Figure 9:**
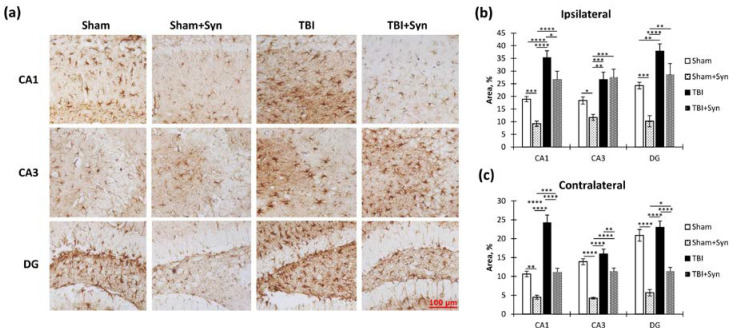
GFAP immunoreactivity within the CA1, CA3, and DG hippocampal areas in TBI and synaptamide treatment. (**a**) Representative photomicrographs of GFAP-positive stained CA1, CA3, and DG areas of ipsilateral hippocampal coronal sections. Scale bar: 100 µm. (**b**) GFAP-positive stained area in CA1, CA3, and DG areas of the ipsilateral hippocampus, %. (**c**) GFAP-positive stained area in CA1, CA3, and DG areas of the contralateral hippocampus, %. The results are shown as mean ± SEM of *n* = 10 independent slides (five animals, with two slides from one animal containing four to five sections). Two-way ANOVA followed by Tukey’s post hoc test (* *p* < 0.05, ** *p* < 0.01, *** *p* < 0.001, **** *p* < 0.0001). Another extremely important astroglial marker is vimentin, the level of which in the brain, as with GFAP, is usually increased in TBI. In general, the pattern of vimentin production in our model looked very similar to that of GFAP (Figure 10a). In both the ipsi- and contralateral hippocampus, TBI caused a powerful increase in vimentin immunoreactivity. In the CA1 region of the ipsilateral hippocampus, two-way ANOVA revealed a significant effect of injury [F(1, 36) = 254.6; *p* < 0.0001] but no treatment effect. A similar picture developed in the DG region, where the effect of trauma was significant [F(1, 36) = 78, 66; *p* < 0.0001]. However, in the CA3 region, synaptamide effectively prevented the TBI-induced increase in vimentin immunoreactivity. A significant effect of injury [F(1, 36) = 106.8; *p* < 0.0001] and treatment [F(1, 36) = 24.77; *p* < 0.0001] was found (Figure 10b).

GFAP is the main protein component of glial intermediate filaments, which is known to be markedly increased in TBI. In addition, the blood level of GFAP is used as a biomarker for the severity of brain damage [14]. A week after injury, we found increased GFAP immunoreactivity in both the ipsi- and contralateral hippocampus (Figure 9a). However, in the ipsilateral hippocampus, the drug prevented the development of astrogliosis to a much lesser extent than in the contralateral one, where the immunoreactivity in the treated animals with TBI was lower than in the animals without treatment. In the CA1 region of the ipsilateral hippocampus, two-way ANOVA revealed a significant effect of both injury [F(1, 36) = 64.35; *p* < 0.0001] and treatment [F(1, 36) = 19.09; *p* < 0.0001]. Interestingly, in the synaptamide-treated sham-operated animals, immunoreactivity was two times lower than in the sham-operated animals without treatment (18.88 ± 1.02—“Sham” vs. 9.20 ± 1.02—“Sham + Syn”, *p* = 0.011). In the CA3 region, where the damage was most extensive, a significant effect of injury was found [F(1, 36) = 24.58; *p* < 0.0001], but no treatment effect was revealed. In the DG region, we identified a significant effect of injury [F(1, 36) = 32.16; *p* < 0.0001] and treatment [F(1, 36) = 16.98; *p* = 0.0002] due to the pronounced effect of synaptamide on the level of GFAP in the sham-operated animals (24.26 ± 1.30—“Sham” vs. 10.17 ± 2.18—“Sham + Syn”, *p* = 0.008) (Figure 9b).

In the contralateral hippocampus, synaptamide effectively prevented the TBI-mediated increase in GFAP immunoreactivity. Thus, in the CA1 region, two-way ANOVA revealed a significant effect of injury [F(1, 36) = 82.43; *p* < 0.0001], treatment [F(1, 36) = 75.68; *p* < 0.0001], and injury/treatment interaction [F(1, 37) = 10.04; *p* = 0.0032]. In the synaptamide-treated TBI animals, the GFAP level was significantly lower than in the animals without treatment (24.26 ± 2.00—“TBI” vs. 11.04 ± 1.09—“TBI + Syn”, *p* < 0.0001). In the CA3 region, two-way ANOVA revealed a significant effect of injury [F(1, 36) = 31.42; *p* < 0.0001], treatment [F(1, 36) = 77.03; *p* < 0.0001], and injury/treatment interaction [F(1, 36) = 9.45; *p* = 0.0041]. There were also injury [F(1, 37) = 9.76; *p* = 0.0036] and treatment [F(1, 36) = 116.3; *p* < 0.0001] effects in the DG region. However, the effect of injury only appeared among the animals treated with synaptamide. In the untreated animals, TBI did not affect GFAP immunoreactivity (Figure 9c). The obtained data demonstrated a synaptamide-induced decrease in GFAP immunoreactivity, mainly in the contralateral hippocampus. In the ipsilateral hippocampus, synaptamide only modestly inhibited the increase in GFAP levels, probably due to significant damage and astroglial scar formation.

**Figure 10:**
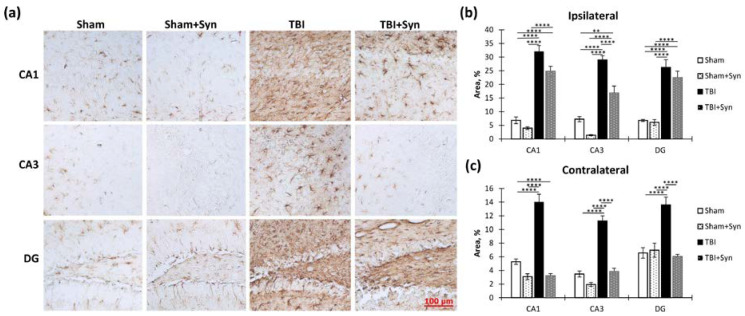
Vimentin immunoreactivity within the CA1, CA3, and DG hippocampal areas in TBI and synaptamide treatment. (**a**) Representative photomicrographs of vimentin-positive stained CA1, CA3, and DG areas of ipsilateral hippocampal coronal sections. Scale bar: 100 µm. (**b**) Vimentin-positive stained CA1, CA3, and DG areas of the ipsilateral hippocampus, %. (**c**) Vimentin-positive stained area in CA1, CA3, and DG areas of the contralateral hippocampus, %. The results are shown as mean ± SEM of *n* = 10 independent slides (five animals, with two slides from one animal containing four to five sections). Two-way ANOVA followed by Tukey’s post hoc test (** *p* < 0.01, **** *p* < 0.0001).

In the contralateral hippocampus, synaptamide not only effectively prevented the TBI-mediated decrease in vimentin immunoreactivity (“TBI”) but also reduced vimentin levels in the sham-operated animals in the CA1 and CA3 regions (“Sham”). Thus, a significant effect of injury [F(1, 36) = 45, 67; *p* < 0.0001], treatment [F(1, 36) = 103.8; *p* < 0.0001,], and injury/treatment interaction [F(1, 36) = 48.48; *p* < 0.0001] was revealed in the CA1 region. In the CA3 region, a significant effect of injury [F(1, 36) = 88.62; *p* < 0.0001], treatment [F(1, 37) = 75, 11; *p* < 0.0001], and injury/treatment interaction [F(1, 36) = 32.29; *p* < 0.0001] was found. In the DG region, a significant effect of injury [F(1, 36) = 13.80; *p* = 0.0006], treatment [F(1, 37) = 18.62; *p* < 0.0001], and injury/treatment interaction [F(1, 36) = 23.06; *p* < 0.0001] was found (Figure 10c).

### 2.6. TBI and Synaptamide Effect on Hippocampal Arc-Protein Production and Adult Neurogenesis

An activity-regulated cytoskeletal gene (Arc or Arg3.1) encodes a protein that is expressed in the postsynaptic density (PSD) and is inherently associated with the regulation of synaptic plasticity and, consequently, memory consolidation processes [15]. In vitro studies have demonstrated that Arc blocking promotes neuronal death after traumatic neuronal injury through necroptosis and apoptosis. However, there are practically no data in the literature on the changes in Arc protein production during TBI in vivo.

**Figure 11:**
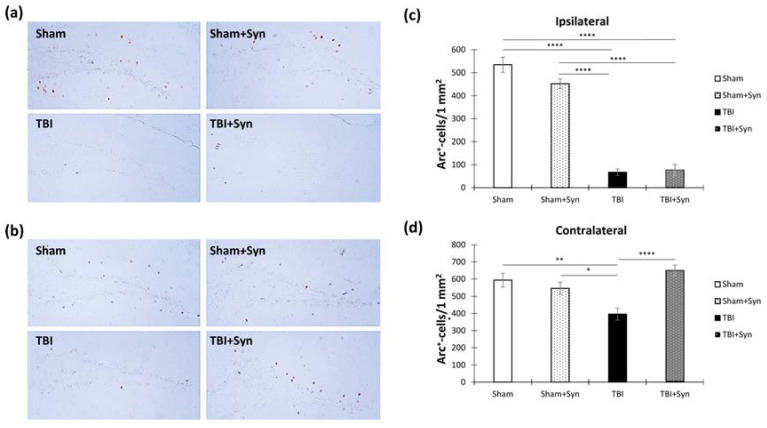
Arc immunoreactivity within the DG hippocampal area in TBI and synaptamide treatment. (**a**) Representative photomicrographs of Arc-positive stained DG area of ipsilateral hippocampal coronal sections. Scale bar: 100 µm. (**b**) Arc-positive stained DG area of the contralateral hippocampus, %. (**c**) Quantification of Arc-positive cells in DG area of the ipsilateral hippocampus, %. The results are shown as mean ± SEM of *n* = 34 independent slides (five animals, with two slides from one animal containing four to five sections). (**d**) Quantification of Arc-positive cells in DG area of the contralateral hippocampus, %. The results are shown as mean ± SEM of *n* = 34 independent slices. Two-way ANOVA followed by Tukey’s post hoc test (* *p* < 0.05, ** *p* < 0.01, **** *p* < 0.0001).

Figure 11a,b show representative images of the ipsi- and contralateral hippocampus DG with immunopositive cells. We found that TBI significantly reduced the number of Arc-positive neurons in the dentate gyrus of the ipsilateral hippocampus compared with the sham-operated animals (534.44 ± 32.77—“Sham” vs. 67.31 ± 13.98—“TBI”, *p* < 0.0001). However, synaptamide therapy was unable to prevent such a dramatic decrease in Arc production (77.24 ± 24.33—“TBI + Syn”). Two-way ANOVA revealed a significant effect of injury [F(1, 36) = 94.17; *p* < 0.0001] (Figure 11c). However, in the contralateral hippocampus, Arc production decreased due to TBI to a lesser extent than in the ipsilateral hippocampus (593.60 ± 39.42—“Sham” vs. 395.95 ± 35.00—“TBI”, *p* = 0.004), and treatment with synaptamide prevented such changes (649.64 ± 31.53—“TBI + Syn”). Two-way ANOVA revealed a significant effect of treatment [F(1, 132) = 6.85; *p* = 0.009] and injury/treatment interaction [F(1, 36) = 14.43; *p* = 0.0002] (Figure 11d).

Adult neurogenesis is a vital aspect of the brain’s neuronal plasticity, which is ongoing throughout life thanks to active stem cells in the hippocampus and olfactory bulbs [16]. Memory and learning are underpinned by the creation of new neurons and their integration into neural networks in the hippocampus [17]. Neurodegenerative diseases and traumatic brain injuries have been associated with alterations in neurogenesis levels. For instance, TBI boosts hippocampal neurogenesis for the first 7 days after injury while simultaneously reducing neuronal survival [18].

**Figure 12:**
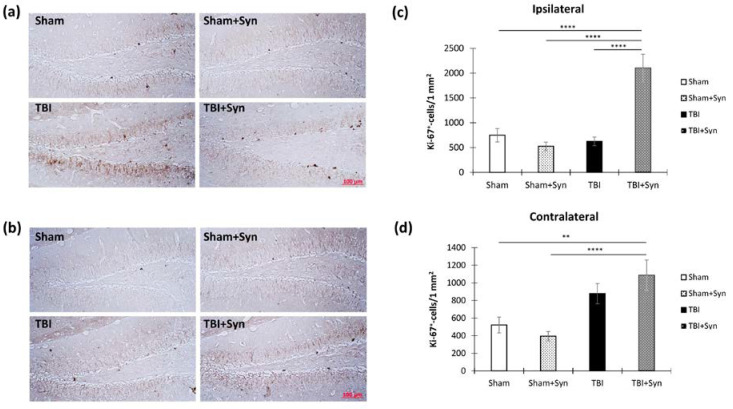
Ki-67 immunoreactivity within the hippocampal DG SGZ in TBI and synaptamide treatment. (**a**) Representative photomicrographs of Ki-67-positive stained DG area of ipsilateral hippocampal coronal sections. Scale bar: 100 µm. (**b**) Ki-67-positive stained DG area of the contralateral hippocampus, %. (**c**) Quantification of Ki-67-positive cells in DG area of the ipsilateral hippocampus, %. The results are shown as mean ± SEM of *n* = 34 independent slides (five animals, with two slides from one animal containing four to five sections). (**d**) Quantification of Ki-67-positive cells in DG area of the contralateral hippocampus, %. The results are shown as mean ± SEM of *n* = 34 independent slices. Two-way ANOVA followed by Tukey’s post hoc test (** *p* < 0.01, **** *p* < 0.0001).

Figure 12a,b show representative images of the ipsi- and contralateral hippocampus DG with Ki-67-positive cells. In our study, adult neurogenesis was assessed by quantifying the proliferation marker Ki-67 in the hippocampal subgranular zone of the dentate gyrus (DG SGZ) and by doublecortin (DCX), a marker of newly formed neurons. However, in our study, 7 days after injury, the number of Ki-67-immunopositive cells in the DG SGZ of the ipsilateral hippocampus did not increase compared to the sham-operated animals. However, the drug significantly increased the number of Ki-67-positive cells in the injured animals (603.97 ± 85.00—“TBI” vs. 2101.24 ± 279.50—“TBI + Syn”, *p* < 0.0001). Two-way ANOVA showed a significant effect of injury [F(1, 36) = 29.88; *p* < 0.001], treatment [F(1, 36) = 23.70; *p* < 0.001], and injury/treatment interaction [F(1, 36) = 43.15; *p* < 0.001]. This result reflected the significant effect of the drug on the number of Ki-67-positive cells in the animals with TBI (Figure 12c). In the contralateral hippocampus, a significant effect of trauma was found [F(1, 36) = 16.17; *p* < 0.0001]. The number of Ki-67-positive cells was significantly higher than in the sham-operated animals (500.70 ± 85.00—“TBI” vs. 1216.23 ± 192.06—“TBI + Syn”, *p* = 0.002) (Figure 12d).

**Figure 13:**
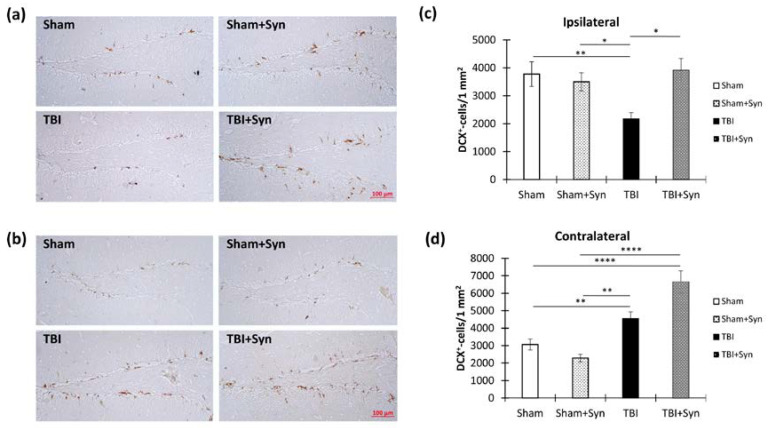
DCX immunoreactivity within the hippocampal DG SGZ in TBI and synaptamide treatment. (**a**) Representative photomicrographs of DCX-positive stained DG area of ipsilateral hippocampal coronal sections. Scale bar: 100 µm. (**b**) DCX-positive stained DG area of the contralateral hippocampus, %. (**c**) Quantification of DCX-positive cells in DG area of the ipsilateral hippocampus, %. The results are shown as mean ± SEM of *n* = 34 independent slides (five animals, with two slides from one animal containing four to five sections). (**d**) Quantification of DCX-positive cells in DG area of the contralateral hippocampus, %. The results are shown as mean ± SEM of *n* = 34 independent slices. Two-way ANOVA followed by Tukey’s post hoc test (* *p* < 0.05, ** *p* < 0.01, **** *p* < 0.0001).

Figure 13a,b show representative images of the ipsi- and contralateral hippocampus DG with DCX-positive cells. Despite the absence of a decrease in the number of proliferating cells in the DG SGZ, the number of newly formed neurons in the animals with TBI without treatment in the ipsilateral hippocampus decreased (3777.247 ± 440.71—“Sham” vs. 2172.05 ± 224.66—“TBI”, *p* = 0.007), which indicated a decrease in the survival of neurons. However, the synaptamide-treated animals with TBI did not show a decrease in the number of DCX-positive cells (3911.69 ± 426.61). Two-way ANOVA revealed a significant effect of injury [F(1, 132) = 4.24; *p* = 0.047], treatment [F(1, 132) = 4.91; *p* = 0.029], and injury/treatment interaction [F(1, 132) = 5.81; *p* = 0.018] (Figure 13c).

An interesting situation was observed in the contralateral hippocampus, where TBI in combination with synaptamide administration significantly increased the number of DCX-positive cells in the DG SGZ (3062.62 ± 310.87—“Sham” vs. 6643.64 ± 643.01—“TBI + Syn”, *p* < 0.0001). Two-way ANOVA revealed a significant effect of injury [F(1, 36) = 47.75; *p* < 0.0001] and injury/treatment interaction [F(1, 36) = 11.57; *p* = 0.0009] (Figure 13d).

### 2.7. Anti-Apoptotic Activities of Synaptamide in TBI

**Figure 14:**
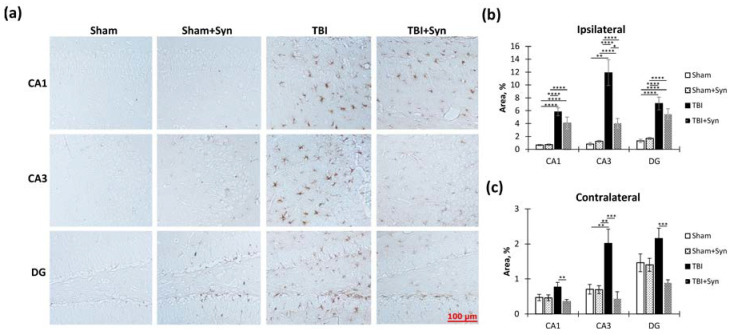
Bcl-2 production within the hippocampus in TBI and synaptamide treatment. (**a**) Representative photomicrographs of Bcl-2-positive stained CA1, CA3, and DG areas of ipsilateral hippocampal coronal sections. Scale bar: 100 µm. (**b**) Bcl-2-positive stained area in CA1, CA3, and DG areas of the ipsilateral hippocampus, %. (**c**) Bcl-2-positive stained area in CA1, CA3, and DG areas of the contralateral hippocampus, %. The results are shown as mean ± SEM of *n* = 10 independent slides (five animals, with two slides from one animal containing four to five sections). Two-way ANOVA followed by Tukey’s post hoc test (* *p* < 0.05, ** *p* < 0.01, *** *p* < 0.001, **** *p* < 0.0001).

**Figure 15:**
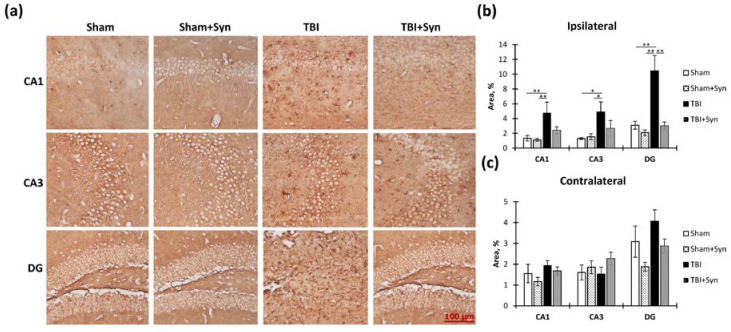
Bad production within the hippocampus in TBI and synaptamide treatment. (**a**) Representative photomicrographs of Bcl-2-positive stained CA1, CA3, and DG areas of ipsilateral hippocampal coronal sections. Scale bar: 100 µm. (**b**) Bcl-2-positive stained area in CA1, CA,3, and DG areas of the ipsilateral hippocampus, %. (**c**) Bcl-2-positive stained area in CA1, CA,3, and DG areas of the contralateral hippocampus, %. The results are shown as mean ± SEM of *n* = 10 independent slides (five animals, with two slides from one animal containing four to five sections). Two-way ANOVA followed by Tukey’s post hoc test (* *p* < 0.05, ** *p* < 0.01).

Figure 14a shows representative photomicrographs of Bcl-2-positive stained CA1, CA3, and DG areas of ipsilateral hippocampal coronal sections. The study of the anti-apoptotic factor Bcl-2 production in the ipsilateral hippocampus revealed a significant TBI-induced increase in Bcl-2 immunoreactivity in the CA1 (0.66 ± 0.23—“Sham” vs. 5.83 ± 0.60—“TBI”, *p* < 0.0001), CA3 (0.82 ± 0.23—“Sham” vs. 11.89 ± 2.00—“TBI”, *p* = 0.009), and DG (1.31 ± 0.25—“Sham” vs. 7.11 ± 0.99—“TBI”, *p* < 0.0001) regions. Two-way ANOVA revealed a significant effect of injury in the CA1 [F(1, 36) = 80.98; *p* < 0.0001], CA3 [F(1, 36) = 83.62; *p* < 0.0001], and DG [F(1, 36) = 64.99; *p* < 0.0001] regions. At the same time, a significant effect of treatment [F(1, 36) = 24.00; *p* < 0.0001] and injury/treatment interaction [F(1, 36) = 29.81; *p* < 0.0001] was revealed in the CA3 region, which indicated the inhibitory effect of synaptamide on the excessive increase in Bcl-2 production (Figure 15b). In the contralateral hippocampus, a more moderate increase in Bcl-2 immunoreactivity was observed, which was expressed only in the CA3 region. Two-way ANOVA revealed a significant treatment effect [F(1, 36) = 6.68; *p* = 0.013], and injury/treatment interaction [F(1, 36) = 4.77; *p* = 0.035] in the CA1 region; injury effect [F(1, 36) = 5.00; *p* = 0.03], treatment [F(1, 36) = 11.71; *p* = 0.001], and interaction [F(1, 36) = 11.38; *p* = 0.001] in the CA3 region; and treatment effect [F(1, 36) = 10.50; *p* = 0.002] and injury/treatment interaction [F(1, 36) = 7.114; *p* = 0.01] in the DG region (Figure 15c). The obtained results indicated that in the damaged hippocampus, synaptamide increased the production of the anti-apoptotic factor Bcl-2, which ultimately prevented neuronal death.

Taken together, the results indicated a stimulatory effect of synaptamide on the protective mechanisms of antioxidant and anti-apoptotic defenses that are impaired due to TBI.

To assess the effectiveness of neuroprotective therapy with synaptamide, in addition to studying the production of the anti-apoptotic factor Bcl-2, we examined the production of the pro-apoptotic factor Bad (Figure 15a). We found a significant increase in the Bad production of TBI in all regions of the ipsilateral hippocampus (Figure 15b). At the same time, synaptamide was unable to prevent apoptotic cell death in the CA1 and CA3 regions of the hippocampus. Two-way analysis of variance revealed a significant effect of injury in both the CA1 [F(1, 36) = 7, 126; *p* = 0.011] and CA3 regions [F(1, 36) = 4.205; *p* = 0.049]. It was only in the DG region that treatment with synaptamide effectively prevented the TBI-mediated decrease in Bad immunoreactivity. Two-way analysis of variance revealed a significant effect of both injury [F(1, 36) = 11.88; *p* = 0.001] and treatment [F(1, 36) = 12.37; *p* = 0.001] as well as an effect of the interaction of factors [F(1, 37) = 7.204; *p* = 0.01]. No significant increase in Bad production was observed in the contralateral hippocampus (Figure 15c).

## 3. Discussion

Traumatic brain injury is a multifaceted phenomenon with a complex pathogenesis that depends on many factors and a variety of consequences in the form of cognitive, sensory, and motor impairments. The negative consequences of TBI are not limited to primary tissue damage but also cause secondary damage resulting from the development of neuroinflammation, oxidative stress, and excitotoxicity [19]. These phenomena in combination lead to the destruction and death of neurons not only directly in the focus of damage but also in nearby tissues not affected by mechanical action. One of the most sensitive regions suffering from secondary damage in TBI is the hippocampus, which is involved in learning, memory consolidation, and higher nervous activity [20]. TBI is a trigger for the activation of neuroinflammatory processes in the hippocampus, against which the development of neuropsychic and cognitive disorders occurs long after the injury. The long-term effects of TBI are closely associated with the development of severe mental disorders such as post-traumatic stress disorder (PTSD) [21]. The basis of mental disorders is a decrease in the number of functional neurons, which occurs as a result of their death [22]. Moreover, the death of newly formed neurons predominantly occurs, while mature neurons undergo degenerative changes, including diffuse axonal damage, and there is a decrease in the density of dendrites and dendritic spines [23]. Neurodegenerative processes inevitably lead to a large-scale loss of synapses and intersynaptic contacts, disrupting the synaptic plasticity and functional activity of surviving neurons [24].

We conducted a detailed study on the morphology of dendrites and dendritic spines in hippocampal neurons that survived TBI and evaluated the effect of synaptamide on structural neuronal plasticity. Additionally, we examined the condition of glial cells, neurogenesis features, and the functionality of antioxidant and antiapoptotic systems in the hippocampus during TBI and treatment with synaptamide. Our findings revealed a significant reduction in the length and branching of dendrites in both pyramidal and granular hippocampal neurons due to TBI. However, synaptamide prevented the degeneration of CA1 and CA3 apical dendrites and DG granular neuron dendrites, preserving their length and number of branches. Moreover, in the CA1 region, synaptamide prevented the reduction in dendritic spine density associated with TBI while significantly increasing the density of spines in the CA3 region and dentate gyrus, regardless of TBI presence. Interestingly, synaptamide in the absence of injury had a marked effect on neuronal morphology. For example, in the CA3 region, the studied drug increased the total length of dendrites and the number of dendritic spines in the sham-operated animals. Moreover, in the dentate gyrus, the drug increased the number of dendritic spines in the animals without TBI. Our analysis of microglia activity largely explained the neuroprotective effects of synaptamide, which has been confirmed to have anti-inflammatory properties in in vitro and in vivo studies [25,26,27,28]. Synaptamide is a highly active compound that belongs to the N-acyl ethanolamines of the fatty acids group. It is an endogenous metabolite and mediator of the central nervous system. Synaptamide has been found to play a crucial role in neuroprotective mechanisms that are activated in various pathological conditions, including neuropathic pain [27,28] and neuroinflammatory processes in the CNS [25,26]. Using cell cultures of mouse embryonic hippocampus, synaptamide has been shown to stimulate neurite outgrowth, the production of synaptic proteins, and synaptogenesis. Moreover, synaptamide exerts these effects at much lower concentrations than docosahexaenoic acid, the metabolic precursor of synaptamide [29,30]. It appears that utilizing drugs with anti-inflammatory properties may hold significant potential, as the underlying disease process is primarily driven by destructive mechanisms related to oxidative stress and excitotoxicity resulting from neuroinflammation.

Immediately following a traumatic brain injury (TBI), there are complex molecular and cellular processes that cause neuroinflammation. This involves the activation of various pro-inflammatory cytokines such as interleukin (IL)-1β, tumor necrosis factor (TNF), IL-6, and chemokines (CCL2, IL-8/CXCL2), as well as other inflammatory mediators (cell adhesion molecules (CAMs), prostaglandins, complements, lysophospholipids). These factors work together to create a cascade of responses that contribute to the further activation of other factors [31,32]. After an injury, the peak of synthesis for pro-inflammatory mediators typically occurs between 2 and 24 h. Following this peak, the synthesis of anti-inflammatory mediators begins to alleviate immune activation. The resulting inflammatory environment triggers the activation of resident glial cells, as well as the extravasation of neutrophils and subsequently macrophages, which can continue for several days [33]. The use of the universal microglia/macrophage marker Iba-1 allowed us to detect a significant increase in microglial/macrophage activity within the hippocampus in TBI [34]. However, synaptamide was unable to reduce the activity of microglia/macrophages in the CA1 region of the damaged hippocampus, and in the dentate gyrus it generally increased the expression of Iba-1 compared to the “TBI” group. To explain this phenomenon, we resorted to an enzyme immunoassay, which allowed us to determine the spectrum of pro- and anti-inflammatory factors produced in the hippocampus, which indicates a predominant microglial phenotype. As a result, we found that the synaptamide-induced increase in Iba-1 immunoreactivity in TBI was accompanied by the upregulation of the anti-inflammatory cytokine IL-10 and the anti-inflammatory M2 microglia marker CD206. At the same time, synaptamide reduced the hippocampal production of the pro-inflammatory cytokine IL-1β, but not IL-6, mainly in the sham-operated animals. The data obtained indicated that synaptamide in TBI promoted the M2 polarization of microglia with a shift in the profile of secreted cytokines towards the anti-inflammatory side. However, technical limitations made it difficult to unambiguously identify specific microglial phenotypes. A recent review compared the results of studying the distribution of M1/M2 markers in TBI [35]. These data should be interpreted with caution, as most studies use mixed cell populations (whole brain homogenate) rather than isolated microglia to specifically study transcriptomic factors. There is an ongoing debate regarding the polarization of microglia in accordance with the M1/M2 macrophage classification. However, compelling evidence indicates that microglial cells undergo continuous changes, resulting in a mixed M1/M2 phenotype [36].

As one of the most abundant cells in the brain, astrocytes contribute significantly to the TBI-associated inflammatory response [37]. Astrocytes play a crucial role in maintaining homeostasis by regulating the influx of immune blood cells and sequestering excess fluid. They also offer metabolic support to neurons while preserving the integrity of the BBB. In case of an injury, astrocytes respond promptly by activating glial fibrillar acid protein (GFAP), vimentin, and S100, and by migrating to the injury site, while also actively proliferating [38]. In our study, we investigated the activity of astrocytes in trauma and treatment immunohistochemically using GFAP and vimentin markers. The data obtained indicated a synaptamide-induced decrease in GFAP immunoreactivity, predominantly in the contralateral hippocampus. In the CA3 area of the ipsilateral hippocampus, synaptamide only modestly inhibited GFAP elevation, probably due to significant damage and astroglial scar formation. The glial scar essentially encloses the damaged area to prevent the migration of inflammatory cells, thereby limiting the spread of neurotoxins to unaffected areas of the brain [39]. It is worth noting that there was some regional specificity regarding the effect of synaptamide treatment on degenerative changes in the dendrites of pyramidal neurons. Obviously, synaptamide was unable to prevent degenerative changes in the basal dendrites of the CA3 region, including a decrease in the number of dendritic spines, which was probably also associated with excessive damage to this region.

Interestingly, the studied drug significantly reduced GFAP production, predominantly in the sham-operated animals. These data, together with the data on the behavior and analysis of neuronal morphology, indicated the activity of the drug in the absence of TBI. A similar picture is observed when studying the immunohistochemical distribution of vimentin, which is produced in extremely small amounts under normal conditions, but its level increases significantly in pathology [40,41]. Research indicates that GFAP and vimentin serve similar roles in the formation of glial scars [40]. Extensive research is necessary to fully understand this matter, but it can be inferred that synaptamide’s impact on astrocytic activity following an injury is responsible for the drug’s neuroprotective abilities. Previous studies have indicated that astrocytes play a crucial role in promoting adult neurogenesis by enhancing neuronal survival [42] and promoting the proliferation of neural stem cells in the hippocampus [43]. However, under pathological conditions such as TBI, astrocytes may also inhibit neurogenesis. For example, blocking the production of GFAP and vimentin by astrocytes has been shown to prevent TBI-mediated decreased neurogenesis and impaired axon regeneration [44,45]. In our study, an increase in the number of newly formed neurons in trauma correlated with a decrease in GFAP and vimentin immunoreactivity in the dentate gyrus of the contralateral hippocampus. In the injured hippocampus, we did not observe a decrease in the synaptamide-induced production of astroglial markers, but we did see a stimulating effect of the drug on neurogenesis, mainly during injury. At the same time, synaptamide did not change the intensity of neurogenesis in the sham-operated animals. Previous studies have demonstrated a multidirectional change in the intensity of hippocampal neurogenesis in TBI depending on the type of injury and time points where the measurements were made. Some studies have shown a decrease in neurogenesis after TBI [20], while others, on the contrary, show an increase in the generation of new neurons in response to injury [46]. The effects of changing the level of neurogenesis can also cause many contradictions and carry both adaptive and maladaptive consequences. For example, treatment with VEGFR2 and mTOR antagonists in experimental TBI in mice and rats reduced neurogenesis, but at the same time it reduced the predisposition to seizures and reduced the morphological abnormalities of newly formed neurons [47,48], which indicates an adaptive direction of the TBI-mediated decrease in neurogenesis. The inhibition of neurogenesis in rats and mice led to cognitive impairment and reduced spatial memory [49,50], indicating the importance of generating new neurons to overcome cognitive deficits with a decrease in neuron density due to their death. Our study found that synaptamide had a positive impact on neurogenesis in the dorsal hippocampus in the cases of TBI. This was accompanied by an improvement in spatial working memory, as assessed in the Y-maze. However, we still do not fully understand why administering synaptamide to mice with TBI resulted in increased locomotor activity. At the same time, neurogenesis is far from the only factor that determines the presence of cognitive deficit in TBI. An important aspect of cognitive functioning is the expression of genes that affect memory and learning. For example, an activity-regulated cytoskeletal gene (Arc or Arg3.1) encodes a protein that is expressed in the postsynaptic density (PSD) and is inherently associated with the regulation of synaptic plasticity and hence memory consolidation processes [51]. In vitro studies show that blocking Arc promotes neuronal death after traumatic neuronal injury through necroptosis and apoptosis [52]. The restoration of Arc protein production under the action of synaptamide in the contralateral hippocampus, which we found, is an important factor in maintaining the normal cognitive status of TBI animals.

However, a very important question remains unanswered: how is the effect of synaptamide on the state of neurites and the production of synaptic proteins associated with the regulation of neuronal survival and apoptotic processes. Studies show that the main participants in the processes of apoptosis, such as caspases, pro- and anti-apoptotic proteins of the Bcl-2 family (Bad, Bax, Bcl-2), etc., are localized in neurites and the synaptic endings of neurons [53]. Their presence in dendrites, axons, and synaptic junctions suggests that dendritic abnormalities and a loss of synapses can locally trigger apoptotic processes [54,55]. In turn, apoptotic stimuli can trigger plastic processes in both newly formed and mature neurons. In addition, TBI is known to be a trigger for energy metabolism disorders, which are associated with mitochondrial damage, hypoxia, excitotoxicity, etc. The above factors lead to the release of ROS by mitochondria, the release of cytochrome c, and the active synthesis of proapoptotic proteins [56]. We suggest that the anti-inflammatory and antioxidant activity of synaptamide underlies the neuroprotective properties of the drug. Synaptamide’s anti-inflammatory properties are achieved through a number of mechanisms, including the activation of the peroxisome-proliferator-activated nuclear receptor-α (PPAR-α), the downregulation of NF-kB- and ERK1/2-dependent signaling, and the accumulation of cAMP followed by the phosphorylation of protein kinase A (PKA) and cAMP-response-element-binding protein (CREB) [5]. A functional synaptamide receptor known as GPR 110 has recently been identified. Binding to this receptor causes cAMP accumulation, which in turn contributes to synaptamide’s anti-neuroinflammatory properties [8]. Our theory is that these processes work in concert to avoid neuronal damage and dendritic degeneration and prevent cell death.

## 4. Materials and Methods

### 4.1. Animals

The study involved 3-month-old male C57Bl/6 mice reared in the vivarium of the National Scientific Center for Marine Biology, Far Eastern Branch of the Russian Academy of Sciences, Vladivostok, Russia. The number of mice per cage was 3–4. Mice had free access to water and food and were kept on a 12 h light/dark cycle. The parameters of the vivarium room were as follows: air temperature 23 ± 2 °C and humidity 55 ± 15%. All manipulations and surgical procedures with animals were approved by the Animal Ethics Committee of the National Scientific Center for Marine Biology, Far East Branch of the Russian Academy of Sciences (No. 8/2022) by the Guidelines for the Welfare of Laboratory Animals and Directive of the Council of the European Community 2010/63/EU.

### 4.2. Surgery and Treatment

**Figure 16:**
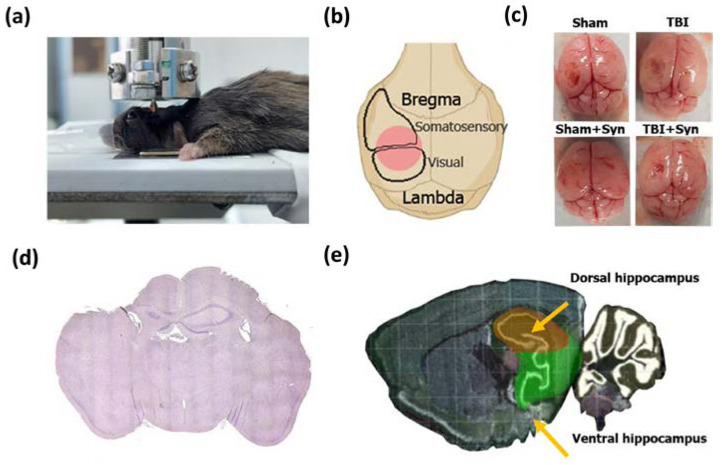
Characteristics of the TBI experimental model. (**a**) The position of the mouse in the laboratory setup for the induction of TBI. (**b**) Localization of the formed cranial window. (**c**) Image of the damaged area 7 days after surgery. (**d**) The area of damage on the hematoxylin-eosin-stained sections was the “TBI” group. (**e**) The region of the hippocampus was selected for evaluation of neurodegenerative processes in the Sholl analysis (a picture from Allen Mouse Brain Atlas, Allen Institute, 615 Westlake Ave North, Seattle, WA, USA).

For the TBI formation in mice, we used the weight-drop injury model (WDI) [57]. This model is a commonly accepted model of TBI representing a weight drop with a given height onto the exposed animal dura. TBI induction was carried out using an IMP-1020 device (Shanghai TOW Intelligent Technology, Shanghai, China) (Figure 16a).

Before the start of the experiment, animals were anesthetized with 4.5% isoflurane in 100% oxygen through a nose cone using an inhalation anesthesia machine (VetFlo™, Kent Scientific Corporation, Torrington, CT, USA). After reaching the stage of deep anesthesia, a skin incision was made on the head along the midline, and the skull was exposed. Before the formation of TBI, a craniotomy was performed with a formed cranial window measuring 4 × 4 mm in the left hemisphere in the middle between bregma and lambda above the area of the visual and somatosensory cortex (Figure 16b). The mouse was placed on an aluminum plate in such a way that the weight was located directly above the cranial window. To form a TBI, a weight of 20 g was lifted with a nylon line to a height of 10 cm and dropped vertically through a guide tube, providing the necessary force of impact on the open brain. After the surgery, the skin over the wound site was treated with an antiseptic and stitched. In the sham-operated groups, craniotomy was performed followed by skin suturing but without TBI. A homeothermic blanket was used to keep the body’s temperature stable. The mortality rate of the mice was zero. Immediately post-surgery, either a synaptamide emulsion or water was administered to animals in vehicle-treated groups. Synptamide emulsion was prepared by mixing synaptamide with water to reach a concentration of 25 mg/mL with constant vortexing (V-32, Biosan, Riga, Latvia). Ethanol was used as an emulsion stabilizer, the amount of which was 1.5% of the injected volume. A similar amount of ethanol was added to the vehicle solution for Veh-treated animals. Synaptamide was injected subcutaneously at a dose of 10 mg/kg.

A total of 56 mice participated in the experiment, which was divided into 4 groups: “Sham”—sham-operated vehicle-treated mice (*N* = 14), “Sham + Syn”—sham-operated synaptamide-treated mice (*N* = 14), “TBI ”—TBI-induced vehicle-treated mice (*N* = 14), and “TBI + Syn”—TBI-induced synaptamide-treated mice (*N* = 14). The amount of drug or vehicle solution administered to one animal was 100 µL, and the duration of administration was 7 days from the day of surgery. Synaptamide was obtained in the authors’ laboratory according to the method described earlier [58].

### 4.3. Behavioral Studies

Behavioral experiments were performed during the light cycle between 7 a.m. and 7 p.m. Behavioral apparatuses were thoroughly cleaned after each animal to minimize olfactory cues. Before the experiment, the mice were left for 2 h in a home cage in the room where the experiments were carried out. Elevated plus maze testing was performed on day 6 after surgery, and Y-maze testing was performed on day 7.

#### 4.3.1. Elevated plus Maze

The experiment used an elevated plus maze (Panlab/Harvard Apparatus, Holliston, MA, USA) consisting of 2 closed arms (height: 15 cm, length: 30 cm, width: 5 cm) and 2 open arms (height: 1 cm, length: 30 cm, width: 5 cm). The mouse was placed on the central platform, leaving it free to move and explore the arms. A video camera with a video-tracking system and the SMART 3.0 software (Panlab/Harvard Apparatus, Holliston, MA, USA) were used to track the mouse’s behavior. The time spent by the mouse in the central part and the open and closed arms of the maze, as well as the number of entrances to the zone of the labyrinth, was tracked and recorded. The behavioral deficits identified in this test were interpreted as changes in the neural defense systems that make the animal either more avoidant or more risk-averse. An increase in the number of entries and time spent in the open arms indicated a high-risk appetite.

#### 4.3.2. Y-Maze

To test working spatial memory, we used a Y-maze consisting of acrylic glass with 3 identical arms. The arm parameters were as follows: length of 30 cm, width of 10 cm, and height of 20 cm. At the beginning of testing, the mouse was placed in the center of the maze, allowing it to move freely and explore the maze. Working spatial memory was expressed by the coefficient of spontaneous alternation, depending on the sequence of entrances to the arms of the maze. The presence of all 4 paws of the mouse in the arm of the maze was considered as an entry criterion. To calculate the coefficient of spontaneous alternation, the following formula was used: Ks = R/A (1), where Ks is the frequency of spontaneous alternations, R is the number of consecutive entries into 3 non-repeating arms, and A is the total number of possible alternations.

### 4.4. Golgi–Cox Staining

The histological Golgi–Cox staining technique was used to visualize hippocampal neurons and subsequently obtain morphometric data. The animals were euthanized on the 7th day after the surgery. To achieve this, mice were anesthetized with isoflurane using a rodent anesthetic vaporizer (VetFloTM, Kent Scientific Corporation, Torrington, CT, USA). Then, decapitation was carried out, and the brain was quickly removed and washed with 0.1 M PBS (+4 °C). An extracted brain on day 7 after surgery is shown in Figure 16c. For visual evaluation of the damaged area, 10 μm thick sections were made, followed by hematoxylin-eosin staining (Figure 16d). The brain was placed in a pre-prepared solution, and all subsequent staining procedures were performed according to the manufacturer’s instructions for the FD Rapid GolgiStainTM kit (FD NeuroTechnologies, Ellicott City, MD, USA). A cryomicrotome (HM 550; Thermo Scientific, Waltham, MA, USA) was used to make 100 µm sections. After further processing, staining, and dehydration, sections on gelatin-coated slides were embedded in H-5000 histological medium (Vector Laboratories, Burlingame, CA, USA).

### 4.5. Sholl Analysis

To assess the state of the dendritic tree of hippocampal neurons, we used sagittal sections of the ipsilateral hippocampus (Figure 16e). Taking into account the fact that part of the hippocampus was destroyed during TBI, we chose for analysis the lateral part of the dorsal hippocampus, where neurons, although having undergone degenerative changes, were not destroyed. At the same time, in the medial part of the dorsal hippocampus, the destruction of neurons was much more fatal, and, therefore, the analysis in this area was difficult. The ImageJ (NIH, Bethesda, MD, USA) was used for image pre-processing and subsequent morphometric studies. For dendritic tracing, a plugin was used (NeuronJ: An ImageJ Plugin for Neurite Tracing and Analysis. Available online: http://www.imagescience.org/meijering/software/neuronj/ (accessed on 20 April 2023)). To perform the Sholl analysis, a plugin was used (Sholl Analysis. Available online: https://imagej.net/plugins/sholl-analysis (accessed on 20 April 2023)). We chose a single neuron as an analytical unit. Per group, there were four animals. The analysis was performed on three well-stained neurons obtained from one animal.

### 4.6. Immunohistochemical Studies

The brain was removed for the immunohistochemical studies on the 7th day after TBI induction. For this, animals were anesthetized with isoflurane using a veterinary vaporizer (VetFlo™, Kent Scientific Corporation, Torrington, CT, USA). After the animals reached the stage of deep anesthesia, transcardial perfusion was performed sequentially with phosphate buffer solution and 4% paraformaldehyde solution (pH 7.2, 4 °C). After that, the brain was removed from the skull and immersed in a 4% paraformaldehyde solution for 12 h for fixation. Next, the samples were washed with a phosphate buffer solution (pH 7.2) and embedded in paraffin blocks. After paraffin blocks were obtained, 10 µm thick coronal sections were made using a rotary microtome (Leica RM 2245, Wetzlar, Germany). At the first stage of immunohistochemical staining, the antigen was unmasked by incubation in 10 mM citrate buffer, pH 6, at 80 °C for 20 min. Endogenous peroxidase activity was blocked using 0.3% hydrogen peroxide solution for 5 min. The preparations were then incubated for 1 h in 5% BSA in PBS to avoid non-specific antibody binding. Treatment with primary antibodies was carried out overnight at 4 °C. Treatment with the secondary antibody anti-rabbit PI1000, 1:100 (Vector Laboratories, Burlingame, CA, USA), labeled with peroxidase was carried out the next day for 2 h. This was followed by a reaction with the ImmPACTTM DAB peroxidase substrate chromogen (SK-4105; Vector Laboratories), washing in PBS, and mounting in VectaMount histological medium (H-5000, Vector Laboratories, Burlingame, CA, USA) (H-5000; Vector Laboratories). The following types of antibodies were used for the study: anti-Iba-1 (rabbit polyclonal, 1:500; ab108539), anti-GFAP (rabbit polyclonal, 1:1000; ab7260), anti-Ki-67 (rabbit polyclonal, 1:1000; ab15580), anti-vimentin (rabbit monoclonal, 1:500; ab92547), anti-Ki-67 (rabbit polyclonal, 1:1000; ab15580), anti-doublecortin (rabbit polyclonal, 1:1000; ab18723), anti-Arc (rabbit monoclonal, 1:100; ab183183), anti-Bcl-2 (rabbit polyclonal, 1:100; ab59348), and anti-Bad (rabbit monoclonal, 1:1000; ab32445) (all from Abcam, Cambridge, MA, USA). Images were obtained using a Zeiss Axio Imager microscope with an AxioCam 503 camera and the AxioVision (Zeiss, Münster, Germany). Image processing was performed using the ImageJ NIH (Bethesda, MD, USA). Image processing included the following steps: image conversion to 8-bit format, background removal (rolling ball radius = 50), and binarization. To assess the state of microglia, astroglia, and the production of apoptotic markers, the area of interest was isolated, and the % fraction of the immunostained area was calculated. Every eight slices were used to evaluate one marker. As an analytical unit, one slide was used, including five sections from one animal. Two glasses were taken from each animal for analysis.

### 4.7. ELISA

ELISA was used to assess the production of CD206, IL-10, IL-1β, IL-6, and synaptophysin proteins. Animals were deeply anesthetized with isoflurane using a veterinary vaporizer (VetFlo™, Kent Scientific Corporation, Torrington, CT, USA). The brain was quickly removed from the skull, and the ipsi- and contralateral hippocampi were isolated separately, frozen in liquid nitrogen, and placed in a freezer at t = −70 °C for storage. For analysis, hippocampi were homogenized in a solution of the following composition: 100 mM Tris, pH 7.4, 150 mM NaCl, 1 mM EGTA and 1 mM EDTA, 1% Triton X-100, 0.5% sodium deoxycholate, and a mixture of protease inhibitors (cOmplete™, Sigma-Aldrich, Bellefonte, PA, USA). The resulting homogenate was incubated on ice for 15 min, centrifuged (16,000× *g*, 30 min, +4 °C), and the supernatant was collected. For the enzyme immunoassay of cytokines IL-10, IL-1β, and IL-6, the following ready-made ELISA kits were used according to the manufacturer’s instructions: Mouse IL-10 ELISA Kit (ab100697), Mouse IL-1β ELISA Kit (ab197742), and Mouse IL-6 ELISA Kit (ab222503) (all from Abcam, Cambridge, MA, USA). For the enzyme immunoassay detection of CD206 and synaptophysin production, the following primary antibodies were used: anti-CD206 (rabbit polyclonal, 1:1000; ab64693) and anti-synaptophysin (rabbit polyclonal, 1:5000; ab32594). For analysis, the resulting supernatants were diluted with bicarbonate–carbonate coating buffer (100 mM, 3.03 g Na_2_CO_3_, 6.0 g NaHCO3, 1000 mL distilled water, pH 9.6) to reach a concentration of 20 g/mL. Next, the samples were placed in the wells of a PVC microtiter plate (M4561-40EA, Greiner, Kremsmünster, Austria) and left overnight at 4 °C. The next day, the solution was removed from the wells, and the plate was washed 4 times with 300 µL of ELISA wash buffer. To block nonspecific binding, 5% milk powder was used, which was added in samples of 100 µL to each well and left overnight. Next, the wells were washed, and 100 µL samples of diluted primary antibodies were added to each and left overnight at 4 °C. After washing, 100 μL samples of peroxidase-labeled secondary antibodies (1:500, PI-1000-1, Vector Laboratories, San Francisco, CA, USA) were added to the wells of the plate and incubated for 2 h at room temperature. After washing, 50 µL of TMB (3,3′,5,5′-tetramethylbenzidine, SK-4400, Vector Laboratories, San Francisco, CA, USA) was added to the wells and incubated for 30 min until the desired degree of staining appeared. Next, 50 μL of stop solution (1 N hydrochloric acid) was added to the wells, giving the formation of a yellow color. Absorbance was measured at 450 nm using an iMark plate spectrophotometer (Bio-Rad, Hercules, CA, USA). A BCA kit (Pierce, Rockford, IL, USA) was used to measure protein concentration.

### 4.8. Evaluation of Superoxide Dismutase Activity

Animals were deeply anesthetized with isoflurane using a veterinary vaporizer (VetFlo™, Kent Scientific Corporation, Torrington, CT, USA). The brain was quickly removed from the skull, and the ipsi- and contralateral hippocampi were isolated separately, frozen in liquid nitrogen, and placed in a freezer at t = −70 °C for storage. For analysis, hippocampi were homogenized in a solution of the following composition: 100 mM Tris, pH 7.4, 150 mM NaCl, 1 mM EGTA and 1 mM EDTA, 1% Triton X-100, 0.5% sodium deoxycholate, and a mixture of protease inhibitors (cOmplete™, Sigma-Aldrich, Bellefonte, PA, USA). The resulting homogenate was incubated on ice for 15 min, centrifuged (16,000× *g*, 30 min, +4 °C), and the supernatant was collected. We used a Superoxide Dismutase Activity Assay Kit for the analysis according to the manufacturer’s instructions (CS0009-1KT, ™, Sigma-Aldrich, Bellefonte, PA, USA). Absorbance was measured at 450 nm using an iMark plate spectrophotometer (Bio-Rad, Hercules, CA, USA). A BCA kit (Pierce, Rockford, IL, USA) was used to measure protein concentration.

### 4.9. Statistical Analysis

The results of the study are presented as mean values ± standard error of the mean. The Shapiro–Wilk test was used to determine the normal distribution of the data. Data were analyzed using two-way ANOVA analysis followed by Tukey’s post hoc multiple comparison tests. For histological studies, 1 neuron was used as an analytical unit (12 neurons per group). For immunohistochemical studies, 1 slide containing 4–5 sections was used as an analytical unit (10 slides per group, with 2 slides from 1 animal). For ELISA, 1 well of a microplate was used as a unit (10 wells per group, with 2 wells from 1 animal). The significance level was set at *p* < 0.05. The Microsoft Excel (Microsoft, Redmond, WA, USA) was used for all statistical tests.

## 5. Conclusions

We performed an explorative study of the pharmacological activity of synaptamide using a mouse TBI model, which allowed us to draw several conclusions about the prospects for further study of this compound. We studied in detail the effect of synaptamide on TBI-associated neurodegenerative changes in the mouse hippocampus. Synaptamide was found to inhibit the TBI-associated decrease in the length and degree of branching of pyramidal and granular neurons’ dendrites in selected hippocampal regions. In addition, synaptamide was effective in preventing the TBI-associated reduction in spine density. A complex study of the microglial activity and production of pro- and anti-inflammatory factors within the hippocampus led to the conclusion that TBI was accompanied by a dynamic change in the production of pro- and anti-inflammatory cytokines and M1-microglia polarization. The data obtained indicate that synaptamide in TBI promoted the M2 polarization of microglia, with a shift in the profile of secreted cytokines towards the anti-inflammatory side. The use of synaptamide prevented TBI-associated astroglial hyperactivation. Synaptamide was shown to be active in the regulation of neuronal plasticity, which was expressed in the improvement of neurogenesis changes and the production of the Arc postsynaptic density protein, encoded by the activity-regulated cytoskeletal gene. The improvement in neuronal plasticity was probably associated with the revealed antioxidant and anti-apoptotic activity of synaptamide. The data obtained allow us to conclude that synaptamide is promising for further study as a therapeutic agent to prevent the neurodegenerative consequences of TBI and improve the quality of life.

## 6. Limitations

1. The design of the study allowed us to study the effects of synaptamide in TBI in the short term, but did not address the long-term consequences.
2. In the study of the production of pro- and anti-inflammatory microglia markers, we used mixed cell populations (whole-hippocampus homogenate) rather than isolated microglia to specifically study transcriptomic factors.

## Data Availability

Data and experimental materials used in this study are available upon request.

## References

[1] Maas A.I.R., Menon D.K., Manley G.T., Abrams M., Åkerlund C., Andelic N., Aries M., Bashford T., Bell M.J., Bodien Y.G. (2022). InTBIR Participants and Investigators. Traumatic brain injury: Progress and challenges in prevention, clinical care, and research. Lancet Neurol..

[2] Voormolen D.C., Haagsma J.A., Polinder S., Maas A.I.R., Steyerberg E.W., Vuleković P., Sewalt C.A., Gravesteijn B.Y., Covic A., Andelic N. (2019). Post-concussion symptoms in complicated vs. uncomplicated mild traumatic brain injury patients at three and six months post-injury: Results from the CENTER-TBI study. J. Clin. Med..

[3] Shirazi R.S., Vyssotski M., Lagutin K., Thompson D., MacDonald C., Luscombe V., Glass M., Parker K., Gowing E.K., Williams D.B.G. (2022). Neuroprotective activity of new Δ3-N-acylethanolamines in a focal ischemia stroke model. Lipids.

[4] Ignatowska-Jankowska B.M., Baillie G.L., Kinsey S., Crowe M., Ghosh S., Owens R.A., Damaj I.M., Poklis J., Wiley J.L., Zanda M. (2015). A cannabinoid CB1 receptor-positive allosteric modulator reduces neuropathic pain in the mouse with no psychoactive effects. Neuropsychopharmacology.

[5] Tsuboi K., Uyama T., Okamoto Y., Ueda N. (2018). Endocannabinoids and related N-acyl ethanolamines: Biological activities and metabolism. Inflamm. Regen..

[6] Katz P.S., Sulzer J.K., Impastato R.A., Teng S.X., Rogers E.K., Molina P.E. (2015). Endocannabinoid degradation inhibition improves neurobehavioral function, blood-brain barrier integrity, and neuroinflammation following mild traumatic brain injury. J. Neurotrauma.

[7] Kim H.Y., Spector A.A. (2018). N-Docosahexaenoylethanolamine: A neurotrophic and neuroprotective metabolite of docosahexaenoic acid. Mol. Asp. Med..

[8] Chen H., Kevala K., Aflaki E., Marugan J., Kim H.Y. (2021). GPR110 ligands reduce chronic optic tract gliosis and visual deficit following repetitive mild traumatic brain injury in mice. J. Neuroinflamm..

[9] Bertagna N.B., Dos Santos P.G.C., Queiroz R.M., Fernandes G.J.D., Cruz F.C., Miguel T.T. (2021). Involvement of the ventral, but not dorsal, hippocampus in anxiety-like behaviors in mice exposed to the elevated plus maze: Participation of CRF1 receptor and PKA pathway. Pharmacol. Rep..

[10] Ghafouri S., Fathollahi Y., Javan M., Shojaei A., Asgari A., Mirnajafi-Zadeh J. (2016). Effect of low-frequency stimulation on impaired spontaneous alternation behavior of kindled rats in Y-maze test. Epilepsy Res..

[11] Pan J., Jin J.L., Ge H.M., Yin K.L., Chen X., Han L.J., Chen Y., Qian L., Li X.X., Xu Y. (2015). Malibatol A regulates microglia M1/M2 polarization in experimental stroke in a PPARγ-dependent manner. J. Neuroinflamm..

[12] Ladak A.A., Enam S.A., Ibrahim M.T. (2019). A review of the molecular mechanisms of traumatic brain injury. World Neurosurg..

[13] Lebkuechner I., Wilhelmsson U., Möllerström E., Pekna M., Pekny M. (2015). Heterogeneity of Notch signaling in astrocytes and the effects of GFAP and vimentin deficiency. J. Neurochem..

[14] Abdelhak A., Foschi M., Abu-Rumeileh S., Yue J.K., D’Anna L., Huss A., Oeckl P., Ludolph A.C., Kuhle J., Petzold A. (2022). Blood GFAP as an emerging biomarker in brain and spinal cord disorders. Nat. Rev. Neurol..

[15] Rodríguez J.J., Davies H.A., Silva A.T., De Souza I.E., Peddie C.J., Colyer F.M., Lancashire C.L., Fine A., Errington M.L., Bliss T.V. (2005). Long-term potentiation in the rat dentate gyrus is associated with enhanced Arc/Arg3.1 protein expression in spines, dendrites and glia. Eur. J. Neurosci..

[16] Kempermann G. (2006). Adult Neurogenesis: Stem Cells and Neuronal Development in the Adult Brain.

[17] Semënov M.V. (2019). Adult hippocampal neurogenesis is a developmental process involved in cognitive development. Front. Neurosci..

[18] Redell J.B., Maynard M.E., Underwood E.L., Vita S.M., Dash P.K., Kobori N. (2020). Traumatic brain injury and hippocampal neurogenesis: Functional implications. Exp. Neurol..

[19] Lozano D., Gonzales-Portillo G.S., Acosta S., de la Pena I., Tajiri N., Kaneko Y., Borlongan C.V. (2015). Neuroinflammatory responses to traumatic brain injury: Etiology, clinical consequences, and therapeutic opportunities. Neuropsychiatr. Dis. Treat..

[20] Wang X., Gao X., Michalski S., Zhao S., Chen J. (2016). Traumatic brain injury severity affects neurogenesis in adult mouse hippocampus. J. Neurotrauma.

[21] Iljazi A., Ashina H., Al-Khazali H.M., Lipton R.B., Ashina M., Schytz H.W., Ashina S. (2020). Post-traumatic stress disorder after traumatic brain injury—A systematic review and meta-analysis. Neurol. Sci..

[22] Wohleb E.S. (2016). Neuron–microglia interactions in mental health disorders: “for better, and for worse”. Front. Immunol..

[23] Cacialli P., Palladino A., Lucini C. (2018). Role of brain-derived neurotrophic factor during the regenerative response after traumatic brain injury in adult zebrafish. Neural Regen. Res..

[24] Ricci G., Volpi L., Pasquali L., Petrozzi L., Siciliano G. (2009). Astrocyte-neuron interactions in neurological disorders. J. Biol. Phys..

[25] Park T., Chen H., Kim H.Y. (2019). GPR110 (ADGRF1) mediates anti-inflammatory effects of N-docosahexaenoylethanolamine. J. Neuroinflamm..

[26] Kim H.Y., Spector A.A. (2013). Synaptamide, an endocannabinoid-like derivative of docosahexaenoic acid with cannabinoid-independent function. Prostaglandins Leukot. Essent. Fatty Acids.

[27] Starinets A., Tyrtyshnaia A., Manzhulo I. (2023). Anti-Inflammatory Activity of Synaptamide in the Peripheral Nervous System in a Model of Sciatic Nerve Injury. Int. J. Mol. Sci..

[28] Paton K.F., Shirazi R., Vyssotski M., Kivell B.M. (2020). N-docosahexaenoyl ethanolamine (synaptamide) has antinociceptive effects in male mice. Eur. J. Pain.

[29] Kim H.Y., Spector A.A., Xiong Z.M. (2011). A synaptogenic amide N-docosahexaenoylethanolamide promotes hippocampal development. Prostaglandins Other Lipid Mediat..

[30] Kim H.Y., Huang B.X., Spector A.A. (2022). Molecular and Signaling Mechanisms for Docosahexaenoic Acid-Derived Neurodevelopment and Neuroprotection. Int. J. Mol. Sci..

[31] Morganti-Kossmann M.C., Semple B.D., Hellewell S.C., Bye N., Ziebell J.M. (2019). The complexity of neuroinflammation consequent to traumatic brain injury: From research evidence to potential treatments. Acta Neuropathol..

[32] Simon D.W., McGeachy M.J., Bayır H., Clark R.S., Loane D.J., Kochanek P.M. (2017). The far-reaching scope of neuroinflammation after traumatic brain injury. Nat. Rev. Neurol..

[33] Ziebell J.M., Morganti-Kossmann M.C. (2010). Involvement of pro- and anti-inflammatory cytokines and chemokines in the pathophysiology of traumatic brain injury. Neurotherapeutics.

[34] Wang G., Zhang J., Hu X., Zhang L., Mao L., Jiang X., Liou A.K., Leak R.K., Gao Y., Chen J. (2013). Microglia/macrophage polarization dynamics in white matter after traumatic brain injury. J. Cereb. Blood Flow Metab..

[35] Donat C.K., Scott G., Gentleman S.M., Sastre M. (2017). Microglial activation in traumatic brain injury. Front. Aging Neurosci..

[36] Italiani P., Boraschi D. (2014). From monocytes to M1/M2 macrophages: Phenotypical vs. functional differentiation. Front. Immunol..

[37] Zhou Y., Shao A., Yao Y., Tu S., Deng Y., Zhang J. (2020). Dual roles of astrocytes in plasticity and reconstruction after traumatic brain injury. Cell Commun. Signal..

[38] Sofroniew M.V. (2015). Astrocyte barriers to neurotoxic inflammation. Nat. Rev. Neurosci..

[39] Wang H., Song G., Chuang H., Chiu C., Abdelmaksoud A., Ye Y., Zhao L. (2018). Portrait of glial scar in neurological diseases. Int. J. Immunopathol. Pharmacol..

[40] Ekmark-Lewén S., Lewén A., Israelsson C., Li G.L., Farooque M., Olsson Y., Ebendal T., Hillered L. (2010). Vimentin and GFAP responses in astrocytes after contusion trauma to the murine brain. Restor. Neurol. Neurosci..

[41] Sarkis G.A., Lees-Gayed N., Banoub J., Abbatielo S.E., Robertson C., Haskins W.E., Yost R.A., Wang K.K.W. (2022). Generation and release of neurogranin, vimentin, and MBP proteolytic peptides, following traumatic brain injury. Mol. Neurobiol..

[42] van Landeghem F.K., Maier-Hauff K., Jordan A., Hoffmann K.T., Gneveckow U., Scholz R., Thiesen B., Brück W., von Deimling A. (2009). Post-mortem studies in glioblastoma patients treated with thermotherapy using magnetic nanoparticles. Biomaterials.

[43] Song H.J., Stevens C.F., Gage F.H. (2002). Neural stem cells from adult hippocampus develop essential properties of functional CNS neurons. Nat. Neurosci..

[44] Wilhelmsson U., Li L., Pekna M., Berthold C.H., Blom S., Eliasson C., Renner O., Bushong E., Ellisman M., Morgan T.E. (2004). Absence of glial fibrillary acidic protein and vimentin prevents hypertrophy of astrocytic processes and improves post-traumatic regeneration. J. Neurosci..

[45] Wilhelmsson U., Pozo-Rodrigalvarez A., Kalm M., de Pablo Y., Widestrand Å., Pekna M., Pekny M. (2019). The role of GFAP and vimentin in learning and memory. Biol. Chem..

[46] Chirumamilla S., Sun D., Bullock M.R., Colello R.J. (2002). Traumatic brain injury-induced cell proliferation in the adult mammalian central nervous system. J. Neurotrauma.

[47] Butler C.R., Boychuk J.A., Smith B.N. (2015). Effects of rapamycin treatment on neurogenesis and synaptic reorganization in the dentate gyrus after controlled cortical impact injury in mice. Front. Syst. Neurosci..

[48] Neuberger E.J., Swietek B., Corrubia L., Prasanna A., Santhakumar V. (2017). Enhanced dentate neurogenesis after brain injury undermines long-term neurogenic potential and promotes seizure susceptibility. Stem Cell Rep..

[49] Blaiss C.A., Yu T.S., Zhang G., Chen J., Dimchev G., Parada L.F., Powell C.M., Kernie S.G. (2011). Temporally specified genetic ablation of neurogenesis impairs cognitive recovery after traumatic brain injury. J. Neurosci..

[50] Sun G., Miao Z., Ye Y., Zhao P., Fan L., Bao Z., Tu Y., Li C., Chao H., Xu X. (2020). Curcumin alleviates neuroinflammation, enhances hippocampal neurogenesis and improves spatial memory after traumatic brain injury. Brain Res. Bull..

[51] Kaizuka T., Takumi T. (2018). Postsynaptic density proteins and their involvement in neurodevelopmental disorders. J. Biochem..

[52] Chen T., Zhu J., Wang Y.H., Hang C.H. (2020). Arc silence aggravates traumatic neuronal injury via mGluR1-mediated ER stress and necroptosis. Cell Death Dis..

[53] Gilman C.P., Mattson M.P. (2002). Do apoptotic mechanisms regulate synaptic plasticity and growth-cone motility?. Neuromol. Med..

[54] Parellada E., Gassó P. (2021). Glutamate and microglia activation as a driver of dendritic apoptosis: A core pathophysiological mechanism to understand schizophrenia. Transl. Psychiatry.

[55] Mattson M.P., Keller J.N., Begley J.G. (1998). Evidence for synaptic apoptosis. Exp. Neurol..

[56] Lewén A., Fujimura M., Sugawara T., Matz P., Copin J.C., Chan P.H. (2001). Oxidative stress-dependent release of mitochondrial cytochrome c after traumatic brain injury. J. Cereb. Blood Flow Metab..

[57] Ma X., Aravind A., Pfister B.J., Chandra N., Haorah J. (2019). Animal models of traumatic brain injury and assessment of injury severity. Mol. Neurobiol..

[58] Tyrtyshnaia A., Bondar A., Konovalova S., Manzhulo I. (2021). Synaptamide improves cognitive functions and neuronal plasticity in neuropathic pain. Int. J. Mol. Sci..

